# Structure Sensitivity of CO_2_ Hydrogenation on Ni Revisited

**DOI:** 10.1021/jacs.3c04284

**Published:** 2023-09-07

**Authors:** Jérôme F. M. Simons, Ton J. de Heer, Rim C. J. van de Poll, Valery Muravev, Nikolay Kosinov, Emiel J. M. Hensen

**Affiliations:** Laboratory of Inorganic Materials and Catalysis, Department of Chemical Engineering and Chemistry, Eindhoven University of Technology, P.O. Box 513, 5600 MB Eindhoven, The Netherlands

## Abstract

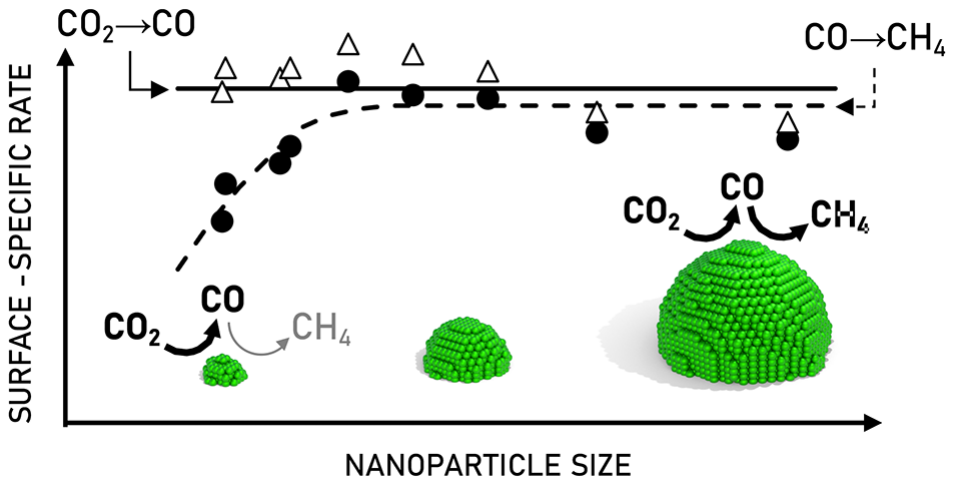

Despite the large number of studies on the catalytic hydrogenation of CO_2_ to CO and hydrocarbons by metal nanoparticles, the nature of the active sites and the reaction mechanism have remained unresolved. This hampers the development of effective catalysts relevant to energy storage. By investigating the structure sensitivity of CO_2_ hydrogenation on a set of silica-supported Ni nanoparticle catalysts (2–12 nm), we found that the active sites responsible for the conversion of CO_2_ to CO are different from those for the subsequent hydrogenation of CO to CH_4_. While the former reaction step is weakly dependent on the nanoparticle size, the latter is strongly structure sensitive with particles below 5 nm losing their methanation activity. Operando X-ray diffraction and X-ray absorption spectroscopy results showed that significant oxidation or restructuring, which could be responsible for the observed differences in CO_2_ hydrogenation rates, was absent. Instead, the decreased methanation activity and the related higher CO selectivity on small nanoparticles was linked to a lower availability of step edges that are active for CO dissociation. Operando infrared spectroscopy coupled with (isotopic) transient experiments revealed the dynamics of surface species on the Ni surface during CO_2_ hydrogenation and demonstrated that direct dissociation of CO_2_ to CO is followed by the conversion of strongly bonded carbonyls to CH_4_. These findings provide essential insights into the much debated structure sensitivity of CO_2_ hydrogenation reactions and are key for the knowledge-driven design of highly active and selective catalysts.

## 1 Introduction

Supported metal nanoparticles are important heterogeneous catalysts for many chemical reactions. Understanding the relation between nanoparticle structure and catalytic activity is essential to maximize reaction rates and minimize side product formation. Increasing the amount of exposed metal atoms is a common approach to improve catalyst performance. However, simply decreasing particle sizes does not always result in a higher activity. The size of nanoparticles governs the arrangement of atoms in surface ensembles, which are represented by mainly edge, corner, terrace, and step-edge sites.^1−4^ Such sites can display very different activities for particular reactions, which is widely known as structure sensitivity.^5^ Therefore, changing the particle size may significantly affect the overall reactivity. A well-known example of such particle size effect is the size-dependent dissociation of the strong π-bonds in N_2_ and CO: surface-specific rates decrease for particle sizes below 5 nm.^6−8^ From surface science studies and density functional theory (DFT) calculations, step edges are regarded as the active sites for the dissociation of π-bonds.^9−12^ The decreased availability of such sites on smaller particles is thought to be responsible for their lower activity in ammonia synthesis and Fischer–Tropsch reactions.^7,12,13^ Establishing the dependence of active site density on nanoparticle size is key for knowledge-driven design of catalysts.

Resolving particle size effects for CO_2_ hydrogenation, which is a promising reaction for upgrading CO_2_ to chemicals and fuels with renewable hydrogen relevant to energy transition scenarios,^14−18^ has recently received substantial interest. Despite significant efforts, the effect of nanoparticle size on the activity and selectivity of CO_2_ hydrogenation to CO and CH_4_ is still debated. Some research groups found increasing surface-specific CO_2_ conversion rates when increasing particle size,^19−24^ while others observed an optimum as a function of particle size,^25−27^ and only few reported that there was no particle size effect at all.^28,29^ Moreover, many studies report high CH_4_ selectivity for small metal nanoparticles, clusters, and even single atoms,^23,25−27,29^ whereas others note that CO is the dominant product for small metal entities, and CH_4_ selectivity increases when increasing the particle size.^20,22,30−32^

These contradictions illustrate the persistent ambiguity about structure–performance relationships for CO_2_ hydrogenation reactions, which implies various mechanistic questions. For the methanation reaction, several studies have identified adsorbed CO as the most abundant surface intermediate.^20,25,31,33^ Typically, the pathways toward CO and subsequently CH_4_ formation are thought to occur on the same surface sites.^33−37^ Consequently, one would expect similar particle size effects as observed for Fischer–Tropsch reactions, particularly when the dissociation of CO is the rate-limiting step. As this mechanistic link with CO hydrogenation is often not reflected by the observed particle size effects, some have suggested that CO_2_ can induce changes of the nanoparticle surface.^25,26,39^ These changes can include oxidation, restructuring, and poisoning, which have been used to interpret the observed particle size effects. Alternatively, parallel reaction pathways have been proposed, where CO_2_ is converted to CH_4_ without the formation of the CO intermediate, typically through surface HCOO or COOH species.^22,32,38^ From the discrepancies between the observed structure–activity relationships and proposed mechanisms, it becomes clear that operando investigations combining accurate kinetic measurements with spectroscopy of surface intermediates are necessary to resolve the mechanism and structure sensitivity of CO_2_ hydrogenation.

In this work, we combined kinetic, isotopic, and spectroscopic tools to analyze the CO_2_ hydrogenation performance of a set of SiO_2_-supported Ni nanoparticles with sizes between 2 and 12 nm. This approach allowed us to determine the structure and evolution of adsorbed surface species and to assess the structural changes of the nanoparticles under reaction conditions. The structure of the nanoparticles was determined by operando X-ray absorption spectroscopy (XAS) and X-ray diffraction (XRD). Time-resolved operando diffuse reflectance infrared Fourier transform spectroscopy (DRIFTS) combined with steady-state isotopic transient kinetic analysis (SSITKA) demonstrated the identity and mechanistic relevance of the adsorbed species during CO_2_ hydrogenation. We found high selectivity toward CH_4_ and constant surface-specific rates for Ni nanoparticles in the range of 5–12 nm. Decreasing the particle size below 5 nm resulted in a sharp decline of CH_4_ formation rates, which was similar to results obtained for CO hydrogenation. Parallel to the decrease in particle size, XRD analysis indicated an increasing number of stacking faults. Other possible structural effects such as oxidation and restructuring were ruled out based on operando XAS and XRD experiments. In contrast to CH_4_ formation, the CO_2_ conversion rates remained unaffected by decreasing particle size, resulting in higher CO selectivity over small nanoparticles. Our results indicate that the formation of CO via the reverse water–gas shift (RWGS) reaction precedes the hydrogenation of CO to CH_4_. With this, we demonstrate the difference in structure sensitivity of RWGS and CO hydrogenation reactions and reveal the role of different active sites in the reaction mechanism of CO_2_ hydrogenation.

## 2 Experimental Methods

### 2.1 Catalyst Preparation

We prepared Ni/SiO_2_ catalysts by incipient wetness impregnation using different concentrations of citric acid to tune the dispersion of the Ni nanoparticles. The SiO_2_ support (X-080, CRI Catalyst Company, 280 m^2^ g^–1^) was dried in air at 110 °C overnight before impregnation. Appropriate amounts of Ni(NO_3_)_2_·6H_2_O (Fischer Scientific, 99%) and citric acid (Merck, >99.5%) were dissolved in deionized water, and this solution was used for impregnation. The impregnated samples were dried in air at 110 °C overnight and subsequently heated at 2 °C min^–1^ in 200 mL min^–1^ 20 kPa O_2_ in He to 400 °C for 4 h. Reduced and passivated samples were obtained by heating the fresh catalysts in 50 mL min^–1^ 10 kPa H_2_ in He to 550 °C at 5 °C min^–1^, holding for 4 h, cooling to room temperature, and exposing the reduced catalysts to 50 mL min^–1^ 2 kPa O_2_ in He for 4 h. The samples are denoted according to the mean area-weighted particle size after H_2_ pretreatment as determined from high-angle annular dark-field scanning transmission electron microscopy (HAADF-STEM), i.e., the Ni2.7 sample has a mean area-weighted particle size of 2.7 nm.

### 2.2 Catalyst Characterization

Ni loadings of the Ni/SiO_2_ catalysts were determined by inductively coupled plasma optical emission spectrometry (ICP–OES) with a Spectro CIROS CCD spectrometer. The particle sizes of the passivated catalysts were examined by HAADF-STEM with a FEI CryoTITAN microscope operating at 300 kV and room temperature. H_2_ chemisorption was performed with a Micromeritics ASAP 2010 instrument. H_2_ uptake was measured after heating the fresh catalysts in flowing H_2_ to 550 °C at 5 °C min^–1^, holding at this temperature for 4 h, and evacuating for 30 min. The H_2_ adsorption isotherms were recorded at 35 °C. The amount of surface Ni was determined by using the linearized form of the Langmuir equation for dissociative adsorption, assuming a Ni:H stoichiometry of 1:1.^40,41^ O_2_ titration was performed with the same instrument by measuring the O_2_ uptake of the catalysts at 400 °C after H_2_ pretreatment and evacuation. Powder XRD was performed with a Bruker Phaser D2 diffractometer using a Cu Kα source. Ni crystallite sizes of the passivated samples were estimated with the Scherrer equation from the integral breadth of the (111) peak, with the Scherrer’s constant *k* taken as unity.^40^ Temperature-programmed reduction by H_2_ (H_2_-TPR) was performed with a Micromeritics ASAP 2929 II instrument. After drying the fresh catalyst at 130 °C in flowing He, the catalyst was heated in diluted H_2_ flow (50 mL min^–1^, 4% H_2_ in He) from 50 to 900 °C at 5 °C min^–1^ while following the H_2_ consumption with a thermal conductivity detector (TCD). Transmission infrared experiments were performed in a Bruker Vertex 70v infrared spectrometer equipped with a DTGS detector. Self-supporting wafers were made by pressing 5–7 mg of the sample in a 13 mm diameter press. Spectra were recorded in a 4000–1000 cm^–1^ range with a resolution of 2 cm^–1^ and averaged over 32 scans. Prior to the CO_2_ adsorption experiments, the samples were pretreated in situ at 550 °C for 4 h in 10 kPa H_2_ in He, evacuated, and cooled down to 220 °C.

Operando XAS was performed at the P65 beamline (Petra III, Hamburg) in transmission mode at the Ni K-edge. A double Si(111) crystal monochromator was used, and the beam size was 0.2 × 1.0 mm. Ni foil was used for energy calibration of each scan. The incoming and transmitted fluxes were measured using ionization chambers. XANES and extended X-ray absorption fine structure (EXAFS) spectra were normalized in the Athena software.^42^ For linear combination fitting of the XANES spectra, a self-developed MATLAB code was used. Fitting of the spectra recorded during the switch from H_2_ to CO_2_ + H_2_ was performed by using the normalized μ(E) spectra of the fresh NiO nanoparticles and Ni nanoparticles after reduction at 550 °C as standards. EXAFS fitting was performed in the R-space with the Artemis software, with fitting ranges of *k* = 2.5–12.3 Å^–1^ and *R* = 1.7–5.1 Å. The amplitude reduction factor S_0_^2^ was determined by fitting the Ni-foil data. A PID controller (Eurotherm) and a custom-built XAS oven were used for temperature control. Fast switching between gases was performed with an in-house developed gas delivery system, consisting of a four-way valve with an electric actuator (VICI), mass flow controllers (Bronkhorst), and pressure controllers (Bronkhorst). An overpressure of 0.25 bar at the outlets of the reactor and a four-way valve was used to minimize the pressure fluctuations during the switches. The outlet of the reactor was connected to a mass spectrometer (Balzer Prisma) to monitor the gas composition during H_2_-pretreatment and CO_2_ hydrogenation steps. In a typical experiment, 25 mg of the catalysts was mixed with 75 mg of sieved (125–250 μm) boron nitride. The catalyst bed was fixed between two quartz wool plugs in a tubular reactor (quartz, i.d. 3 mm) with flattened windows to improve the transmission signal.^43^ The fresh catalysts were heated in 50 mL min^–1^ 10 kPa H_2_ in Ar to 550 °C at 5 °C min^–1^. After cooling to 220 °C, a switch was performed from 50 mL min^–1^ 20 kPa H_2_ in Ar to 50 mL min^–1^ 5 kPa CO_2_ and 20 kPa H_2_ in Ar.

Operando XRD measurements were performed at the ID31 beamline (ESRF, Grenoble) using a beam energy of 75 keV and a size of 380 × 370 μm. Diffraction patterns were collected with a PILATUS3 X CdTe 2M area detector in Debye–Scherrer geometry. Calibration of the detector was performed with a CeO_2_ NIST reference. PyFAI software was used to integrate the 2D diffraction patterns.^44^ The diffraction pattern of the same reactor filled with bare silica support was used for background removal. Gas blowers were used for heating, and the home-made gas delivery system was the same as described above for the operando XAS experiments. The outlet of the reactor was connected to a mass spectrometer (Balzer Prisma) to monitor the gas composition during H_2_ pretreatment and CO_2_ hydrogenation steps. Typically, 20 mg of the catalyst was loaded in a quartz capillary (i.d. 2.8 mm) and fixed with quartz wool plugs. The fresh catalysts were heated in 50 mL min^–1^ 10 kPa H_2_ in Ar to 550 °C at 5 °C min^–1^. After cooling to 220 °C, a switch was performed from 50 mL min^–1^ 20 kPa H_2_ in Ar to 50 mL min^–1^ 5 kPa CO_2_ and 20 kPa H_2_ in Ar. Whole pattern powder modeling (WPPM) was performed using PM2K software.^45^ The instrumental parameters were determined by fitting the pattern of CeO_2_ (NIST 674b) reference material. The lattice parameter, deformation fault probability, crystal size distribution, and Chebyshev background–polynomial parameters were refined during the fitting procedure.

Operando DRIFTS experiments were performed in a Vertex 70v FT infrared spectrometer (Bruker). We used a MCT detector, a mid-IR laser source, and a Praying Mantis accessory and cell (Harrick). In a typical experiment, the bottom of the sample cup (i.d. 6.4 mm) was filled with quartz wool. SiC powder (125–250 μm) was placed on top of the quartz wool layer to ensure optimal gas flow distribution. Approximately 10 mg of the catalyst was placed on top of the SiC layer, with the bottom of the catalyst bed in direct contact with the thermocouple. Gases were introduced at the bottom of the sample cup. The gas composition after the catalyst bed was probed by mass spectrometry (MS) (Balzer Prisma), where the inlet of the MS capillary was positioned close to the catalyst bed (3–4 mm, Figure S8c). This configuration allowed fast gas replacement during switches, with an equal gas hold-up times obtained from both IR and MS. The same custom-built gas delivery system as described above was used for switches between the different gas feeds. The catalysts were heated in 50 mL min^–1^ 10 kPa H_2_ in Ar to 550 °C at 5 °C min^–1^ and subsequently cooled down to 200 °C. Background spectra were recorded while exposing the catalyst to 50 mL min^–1^ 20 kPa H_2_ in Ar flow at 200 °C. The spectra were corrected for the differences in optical pathlength by using the overtone and combination vibrations of silica in the 2100–1800 cm^–1^ region.^46,47^ In addition, the recorded spectra were normalized by the Ni surface area derived from H_2_ chemisorption to account for differences in Ni dispersion. The transient experiments were performed at a lower temperature of 200 °C to increase the differences in residence times between surface species. Rapid-scan mode was used to achieve high temporal resolution. An aperture of 8 cm^–1^ with a resolution of 4 cm^–1^ and a spectral range of 4000–1000 cm^–1^ were used. For the DRIFTS–SSITKA experiments, the response of the ^12^C- and ^13^C-containing species was fitted with a Gaussian function (Figure S12). A self-developed MATLAB script was used to determine the time-dependent fraction of these species after the SSITKA switch.

### 2.3 Activity Measurements

The catalytic activity was measured in a stainless steel plug flow reactor with an internal diameter of 5 mm and a bed length of 80 mm. Appropriate amounts of sieved (125–250 μm) catalysts were diluted with SiC of the same mesh and loaded in the reactor. Prior to the catalytic tests, the fresh catalyst was heated in 50 mL min^–1^ 10 kPa H_2_ in Ar to 550 °C at 5 °C min^–1^, holding for 4 h and cooling to 220 °C. Subsequently, the reduced catalyst was exposed to 50 mL min^–1^ 5 kPa CO_2_ and 20 kPa H_2_ in Ar. The reaction flow composition was determined by online gas chromatography (TRACE 1300 GC, Thermo Scientific) using TCD and FID detectors. The reported activity data was obtained after at least 10 h of steady-state operation. Reaction orders with respect to CO_2_ and H_2_ were determined by, respectively, varying the CO_2_ concentration between 3 and 5 kPa, and the H_2_ concentration between 16 and 24 kPa, whilst adjusting the Ar concentration to keep a constant total flow rate of 50 mL min^–1^. The apparent activation energy was determined by measuring steady-state reaction rates between 216 and 224 °C at intervals of 2 °C. Further details about the thermodynamic equilibria of RWGS and methanation reactions, and mass- and heat-transfer limitations are provided in Notes S4 and S5 in the Supporting Information

SSITKA was used to determine the surface residence time and coverage of the intermediates (see Note S1). A four-way valve (VICI Valco, N4WE) driven by compressed air with a small internal volume (100 μL) enabled a fast switch between gas flows, instantaneously replacing ^12^CO_2_ by ^13^CO_2_ (99% ^13^C, Eurisotop). Ne was added as an inert tracer to account for the gas-phase hold-up in the system. The transients of ^12^CH_4_ (*m*/*z* = 15), ^13^CH_4_ (*m*/*z* = 17), Ne (*m*/*z* = 22), ^12^CO (*m*/*z* = 28), ^13^CO (*m*/*z* = 29), ^12^CO_2_ (*m*/*z* = 44), and ^13^CO_2_ (*m*/*z* = 45) were monitored by an online quadrupole mass spectrometer (ESS, GeneSys Evolution). By following the transient response of the products relative to the Ne tracer, we were able to determine the surface residence time of the intermediates leading to different products.^48^

## 3 Results and Discussion

### 3.1 Structure and Chemical State of Catalysts Prior to Activity Tests

Ni/SiO_2_ catalysts were prepared by incipient wetness impregnation, where citric acid was added to the impregnation solution. Citric acid has been reported to strengthen the interaction of Ni ions with the support and inhibit the redistribution of Ni during drying, resulting in highly disperse Ni nanoparticles after calcination and reduction.^49,50^ By varying the ratio between citric acid and the Ni precursor, we tuned the size of Ni particles while preserving narrow particle size distributions. Since the Ni weight loading had a minor effect on the particle size (Figure S2), we varied the Ni loadings to minimize differences in Ni surface areas between reduced samples. An overview of the physical–chemical properties of the prepared Ni catalysts is presented in Table 1.

**Table 1:** Characteristics of the Prepared Ni/SiO_2_ Catalysts

sample	Ni content (wt %)^a^	citric acid/Ni ratio (−)	*d*_n,STEM_ (nm)^b^	σ_n,STEM_ (nm)^c^	*d*_s_,_STEM_ (nm)^d^	*d*_H_2_ chemisorption_ (nm)	*d*_XRD_ (nm)
Ni2.7	1.7	7.69	2.3	0.6 (27%)	2.7	2.7	
Ni2.8	2.4	1.85	2.5	0.5 (19%)	2.8	2.6	
Ni3.7	3.3	0.91	3.3	0.7 (23%)	3.7	2.9	3.8
Ni3.9	4.8	0.38	3.5	0.8 (23%)	3.9	3.0	3.8
Ni4.8	9.4	0.14	4.2	0.9 (22%)	4.8	5.3	5.3
Ni5.9	7.9	0.10	5.0	1.5 (31%)	5.9	5.1	6.5
Ni7.2	10.0	0.08	6.1	1.7 (28%)	7.2	7.3	7.5
Ni9.0	11.9	0.05	7.5	2.3 (31%)	9.0	10.2	9.1
Ni12.2	14.6	0.03	10.3	2.8 (28%)	12.2	14.2	11.0

a Ni weight loading determined from ICP–OES.

b Mean number-weighted particle size from HAADF-STEM.

c Standard deviation and relative standard deviation with respect to the mean (in brackets) of the number-weighted particle size distribution from HAADF-STEM.

d Mean area-weighted particle size from HAADF-STEM.

**Figure 1:**
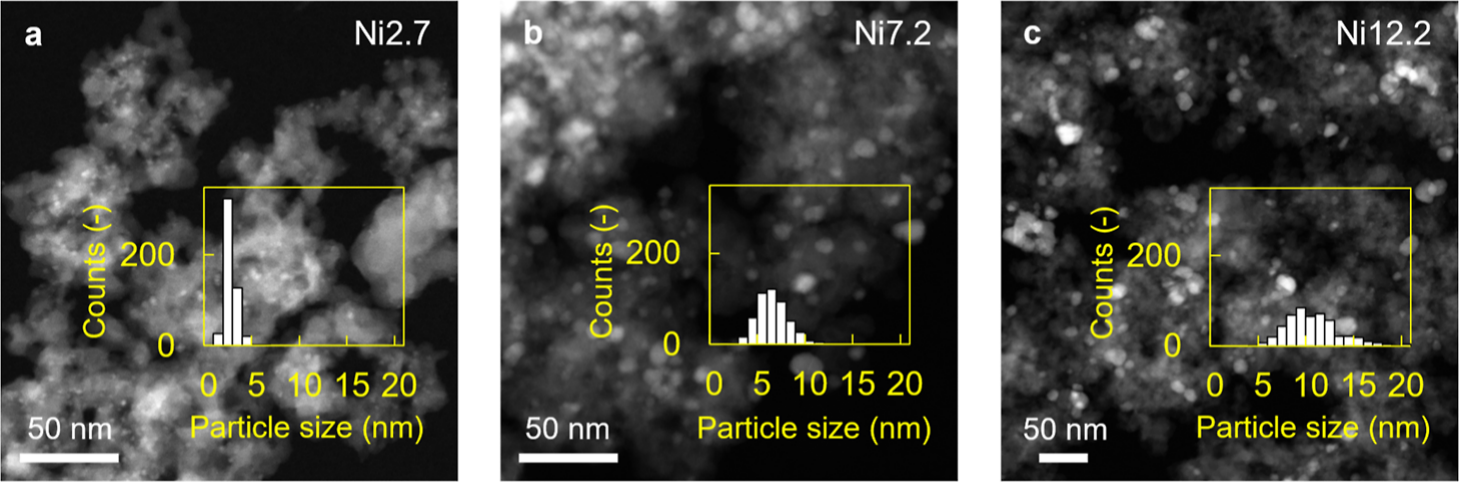
HAADF-STEM images of Ni/SiO_2_ catalysts. Particle size distributions of (a) Ni2.7, (b) Ni7.2, and (c) Ni12.2 catalyst samples after H_2_ pretreatment at 550 °C and passivation by exposure of 2 kPa O_2_ in He at room temperature.

With HAADF-STEM, we found a mean area-weighted particle size of 2.7 nm when using the highest citric acid-to-Ni ratio, while for the lowest ratio, a particle size of 12.2 nm was obtained (Figure 1). For all samples, the standard deviation was 20–30% of the mean particle size. As shown in Table 1, the mean area-weighted particle size values derived from electron microscopy agree well with the particle sizes estimated from H_2_ chemisorption and are comparable to the crystallite sizes determined from XRD. Additional information regarding the characterization of the prepared catalysts can be found in the Supporting Information.

### 3.2 Particle Size Effect on CO_2_ Hydrogenation Performance

The Ni particle size dependence of CO_2_ conversion and CH_4_ formation rates was assessed by measuring the CO_2_ hydrogenation catalytic activity in a fixed-bed reactor at 220 °C. We only observed CO and CH_4_ as reaction products. As it has been previously shown that the conversion level strongly affects CH_4_ selectivity in CO_2_ hydrogenation,^24,32,33^ we kept the conversion for all samples constant in a narrow range of 0.015–0.018 by varying the amount of catalyst loaded. Prior to the activity tests, the catalysts were pretreated in H_2_ at 550 °C to ensure complete reduction of the Ni particles (Figure S3c).

**Figure 2:**
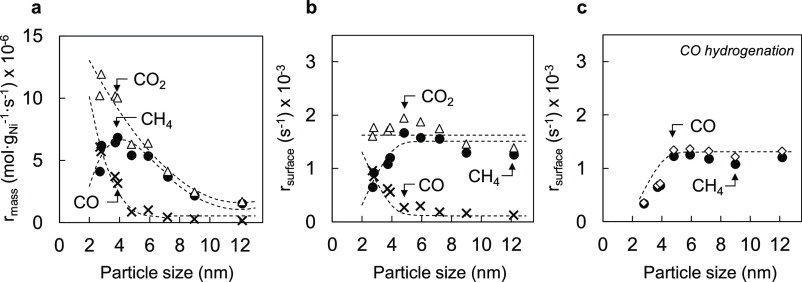
Catalytic performance during CO_2_ and CO hydrogenation. (a) Ni mass-normalized CO_2_ conversion (open triangles), CH_4_ formation rates (solid circles), and CO formation rates (crosses) versus particle size during CO_2_ hydrogenation at 0.015–0.018 CO_2_ conversion (220 °C, 50 mL min^–1^ of 5 kPa CO_2_ and 20 kPa H_2_ in Ar). (b) Ni surface-specific CO_2_ conversion (open triangles), CH_4_ formation (solid circles), and CO formation rates (crosses) rates versus particle size. Surface-specific rates are calculated with Ni dispersion values derived from H_2_ chemisorption. (c) Ni surface-specific CO conversion (open diamonds) and CH_4_ formation (closed circles) rates observed during CO hydrogenation (220 °C, 50 mL min^–1^ 2 kPa CO and 20 kPa H_2_ in Ar). Dashed lines are used to guide the eye.

Figure 2a shows the Ni-weight-normalized CO_2_ conversion and CH_4_ formation rates. Decreasing the particle size from 12.2 to 2.7 nm results in an increase in CO_2_ conversion rates from 1.7 × 10^–6^ to 10.2 × 10^–6^ mol g_Ni_^–1^ s^–1^. This trend can be explained by the increased specific Ni surface area when decreasing the Ni particle size. Similarly, CH_4_ formation rates increase from 1.5 × 10^–6^ to 4.1 × 10^–6^ mol g_Ni_^–1^ s^–1^ when decreasing the size from 12.2 to 2.7 nm. Notably, the CH_4_ formation rate reaches a maximum of 6.8 × 10^–6^ mol g_Ni_^–1^ s^–1^ at 3.9 nm and decreases for smaller particles. This decrease in CH_4_ formation when decreasing the particle size is accompanied by higher CO formation rates. Normalization by the Ni surface area shows that the CO_2_ conversion rate per surface Ni atom is independent of particle size with a value of approximately 1.7 × 10^–3^ s^–1^ for all samples (Figure 2b). In contrast, CH_4_ formation rates of ∼1.6 × 10^–3^ s^–1^ are observed for particles between 5 and 12 nm, and this rate declines to 0.7 × 10^–3^ s^–1^ for the smallest Ni particles in the set. This decrease in CH_4_ formation rate is in line with particle size effects previously observed for CO hydrogenation.^6,12,51^ To assess the similarities between CH_4_ formation from CO_2_ and CO, we also measured the surface-specific rates of CO hydrogenation for the same set of catalysts. As shown in Figure 2c, the overall conversion rates were slightly lower than the ones found for CO_2_ hydrogenation, likely due to CO poisoning (Figure S5c). Still, CH_4_ formation rates as a function of particle size are close to the ones observed during CO_2_ hydrogenation and display the same decrease in surface-specific rates below 5 nm.

The similar dependence of CO_2_ and CO methanation rates on particle size demonstrates that the structural requirements of CH_4_ formation are the same for both reactants. In contrast, surface-specific CO_2_ conversion rates are insensitive to variation in Ni particle size. This result indicates that the structural requirements for the reaction steps in CO_2_ hydrogenation leading to the formation CO and CH_4_ are different.

### 3.3 Nanoparticle Structure during Reaction Conditions

**Figure 3:**
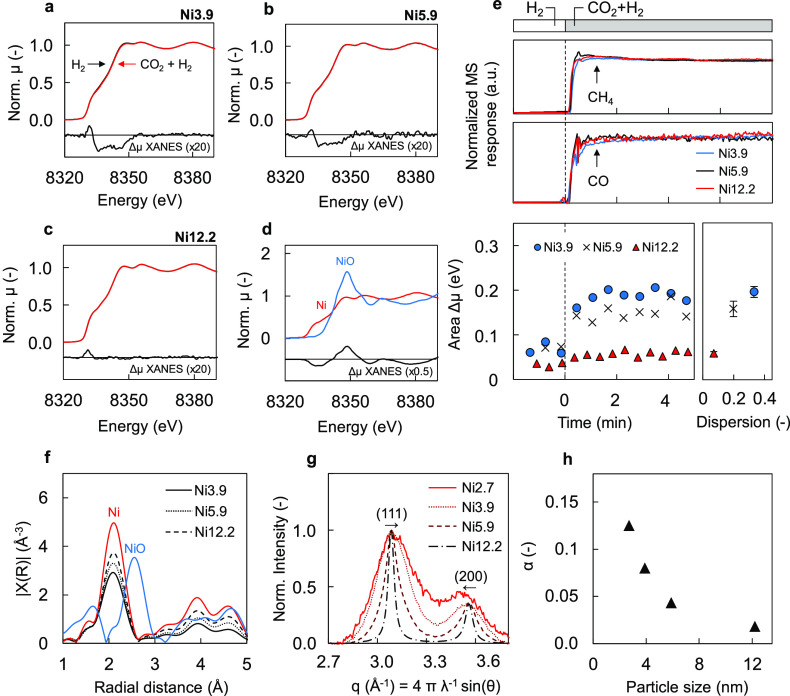
Structure of Ni nanoparticles under reaction conditions. Ni K-edge XANES spectra for Ni3.9 (a), Ni5.9 (b), and Ni12.2 (c) when exposed to H_2_ (black line) or CO_2_ + H_2_ flow (red line) at 220 °C. Δμ XANES results, multiplied by 20, are shown below the spectra. (d) XANES spectra of Ni K-edge for Ni-foil and NiO references with the Δμ XANES spectrum shown at the bottom. (e) MS results and Δμ XANES area during switch from 20 kPa H_2_ to 5 kPa CO_2_ + 20 kPa H_2_ in Ar at 220 °C. Normalized MS response for CH_4_ (*m*/*z* = 15) and CO (*m*/*z* = 28) versus time (top panel). Area of the Δμ XANES features versus time and Ni dispersion (bottom panel). (f) EXAFS R-space plot for Ni3.9 (solid line), Ni5.9 (dotted line), and Ni12.2 (dashed line) after the switch to CO_2_ + H_2_ and for Ni and NiO references. (g) Operando XRD results of different Ni particle sizes when exposed to 20 kPa H_2_ in Ar at 220 °C. (h) Deformation fault probability α versus particle size, as determined from the whole powder pattern modeling.

Previously, particle size effects for CO_2_ and CO hydrogenation have been linked to the oxidation of the Ni surfaces or accumulation of C*/CO* species.^25,52−56^ To determine the effect of CO_2_ hydrogenation on the oxidation state of the Ni particles, operando XAS measurements were performed (Figure 3a–d). For these experiments, the catalysts were pretreated with H_2_ at 550 °C, followed by cooling to 220 °C. Once the temperature was stabilized, we rapidly switched from H_2_ to CO_2_ + H_2_ while continuously recording the XANES spectra. MS results show the rapid formation of CH_4_ and CO after the switch, and stable formation rates were reached quickly (Figure 3e, top panel). Linear combination fitting of the time-resolved XANES using NiO and Ni^0^ references did not show any appreciable oxidation to Ni^2+^ upon exposure to CO_2_ + H_2_ (Figure S7). EXAFS results confirm the absence of NiO formation and major nanoparticle restructuring (Figure 3f). However, careful examination of the spectra revealed subtle differences in the edge shape after the introduction of CO_2_ (Figure 3a–c). Specifically, an increase in the intensity of the low-energy shoulder is observed, accompanied by a shift of the edge to higher energies. For the Ni K-edge, the formally forbidden 1s → 3d transition is possible for tetrahedral or distorted octahedral metal site symmetries.^57,58^ This transition appears at a lower energy than the main edge transition and gains intensity due to the hybridization of 3d and 4p orbitals.^59−61^ As shown in Figure 3e (bottom panel), the changes in the low energy part of the spectra occur rapidly after the switch. Moreover, increasing the overall pressure led to an increase of these changes, while removing CO_2_ from the reaction mixture restored the initial shape of the spectrum (Figure S12c–f). The observed changes in the edge region point at the interaction of Ni species with adsorbed intermediates during CO_2_ hydrogenation. The magnitude of these changes is generally proportional to the Ni dispersion, although slightly diminished for the smallest Ni nanoparticles (Figure 3e, bottom panel). While these results demonstrate the high sensitivity of XANES to changes in surface characteristics of Ni nanoparticles and indicate that the amount of surface intermediates for different particle sizes might be different, they also rule out oxidation of Ni during the reaction as the reason for the observed particle size effect.

To further study the structure of Ni nanoparticles during the reaction, we performed operando XRD experiments. Similar to the XAS experiments, the catalysts were placed in quartz fixed-bed reactors and pretreated with H_2_ at 550 °C before performing the H_2_/CO_2_ + H_2_ switches at 220 °C. Here, no differences in the diffractograms were observed after the introduction of CO_2_, confirming the absence of oxidation or restructuring for all particle sizes (Figure S8). However, differences in the diffraction patterns were observed between the Ni particle sizes (Figure 3g). Besides the expected broadening of diffraction peaks upon decreasing the particle size, a shift to higher *q* values was observed for the (111) peak when decreasing particle size. In contrast, the position of the (200) peak shifted in the opposite direction, which is characteristic for deformation faulting.^62,63^ In many fcc metals, stacking faults are common planar defects where stacking of ABCABC layers in the 111 direction is disturbed. When a deformation fault occurs, the sequence of packing A, B, and C layers is interrupted with one of the atomic planes being skipped in the stacking sequence (e.g., ABCBCABC). In turn, twin faults correspond to the reversing of the stacking sequence (e.g., ABCBACBA). While the effect of stacking faults on specific diffraction peaks may differ,^64,65^ peak position shifts can be attributed to variations in deformation fault probability.^64,66,67^ Here, the excellent signal-to-noise ratio of the synchrotron data allowed detailed analysis of small Ni nanoparticles. To extract microstructural information from the diffractograms, WPPM was performed using the PM2K software package.^45^ With this, the deformation fault probability α could be determined as a function of particle size. More information on the fitting procedure and the results can be found in Note S3. As shown in Figure 3h, the α values for the samples with particle sizes of 5.9 and 12.2 lie around 0.02–0.04 and are similar to values previously reported in the literature.^66,68−70^ When decreasing the Ni particle size, α increases up to a value of 0.13 for 2.7 nm particles. A strong surface strain is typically considered to be the driving force for the formation and growth of stacking faults, which are often found to increase with decreasing nanoparticle size.^71−80^ Several recent studies have highlighted the possible effects of bulk distortion on the catalytic activity of nanoparticles.^81−89^ For example, Tsakoumis et al.^89^ found that Co particles with few crystal defects outperform Co particles with significant lattice defects in terms of CO hydrogenation activity. Nevertheless, the exact impact of bulk distortions on the surface structure of small nanoparticles as postulated here requires further investigations.

### 3.4 Surface Coverages and Kinetic Response of Ni Nanoparticles

To gain an insight into the surface coverage of intermediates during CO_2_ hydrogenation, we applied SSITKA. After reaching steady state under CO_2_ hydrogenation conditions, ^12^CO_2_ in the feed was rapidly replaced by ^13^CO_2_, while Ne was added to account for the gas hold-up time. Due to the kinetic resistance of surface reactions, the transients of the labeled CH_4_ and CO products are expected to be delayed with respect to the inert Ne tracer (Figure S6e). Thus, the difference in residence time between the isotopically labeled products and the tracer yields the mean surface residence time τ. Readsorption effects were corrected for by varying the amount of the catalyst and extrapolating to zero catalyst loading, yielding the corrected mean surface residence time τ_0_.^48^

**Figure 4:**
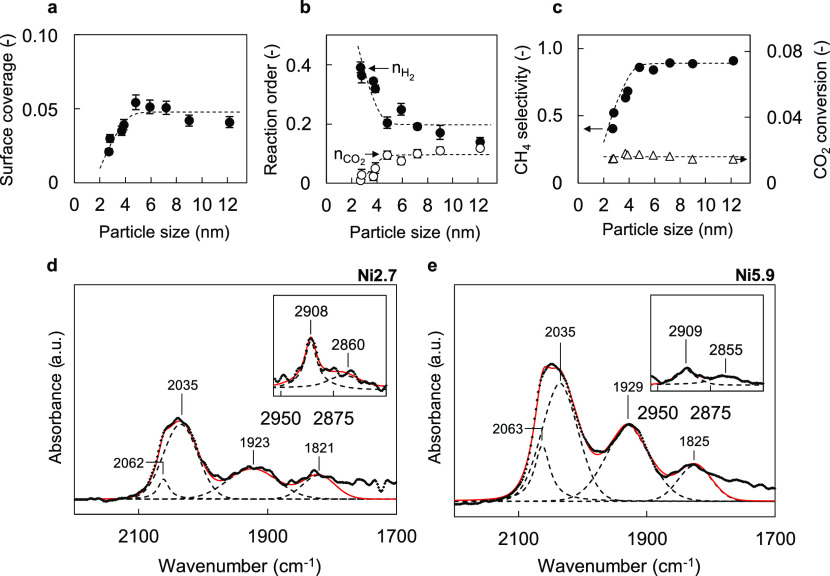
Surface coverages, catalyst performance, and DRIFTS spectra recorded during CO_2_ hydrogenation. (a) Coverage of surface intermediates leading to CH_4_ calculated with τ_0_ values from SSITKA experiments and Ni surface area from H_2_ chemisorption. (b) Reaction orders of H_2_ (solid circles) and CO_2_ (open circles) for CH_4_ formation (16–24 kPa H_2_, 4–6 kPa CO_2_). (c) CH_4_ selectivity (left axis) and CO_2_ conversion (right axis) versus particle size. Dashed lines are used to guide the eye. The constant values of the dashed lines for the 5–12 nm range were based on the general trends observed from our kinetic results. (d) DRIFTS spectra of Ni2.7 during steady-state CO_2_ hydrogenation at 200 °C. The inset displays the spectra in the 2960–2800 cm^–1^ range. (e) DRIFTS spectra of Ni5.9 during steady-state CO_2_ hydrogenation at 200 °C. The spectra are corrected for the differences in optical pathlength by using the overtone and combination vibrations of silica in the 2100–1800 cm^–1^ region. In addition, the spectra are normalized by Ni surface area as determined from H_2_ chemisorption. The same *y*-axis (absorbance a.u.) is used for the Ni2.7 and Ni5.9 samples. For the insets, the same *y*-axis is used for both the samples as well.

From the SSITKA experiments performed during CO_2_ hydrogenation at 220 °C, surface residence times of the intermediates leading to CH_4_ and CO are found to be unaffected by particle size (Figure S6a). τ_0_ was determined to be 32 ± 2 and 16 ± 3 s for CH_4_ and CO intermediates, respectively (Figure S6d). With these values, the coverage of surface intermediates can be calculated, assuming a 1:1 stoichiometry between intermediates and surface Ni atoms.^43^ However, as the surface residence time of CO intermediates only accounts for the intermediates that desorb and leave the reactor in the effluent flow, this residence time does not include the contribution of CO intermediates that may be hydrogenated to CH_4_. Therefore, we will focus on the results of the surface intermediates leading to CH_4_. As shown in Figure 4a, the coverage of CH_4_ intermediates initially increases from 0.025 to 0.05 with increasing particle size and remains constant for particle sizes above 5 nm. For the case of CO hydrogenation, a τ_0_ value of 32 ± 4 s was determined for CH_4_ intermediates, resulting in similar coverages as observed during CO_2_ hydrogenation (Figure S6f). Apparent activation energy values for CH_4_ formation from CO_2_ and CO hydrogenation confirm the similarities in methanation kinetics, with respective values of 91 ± 2 and 94 ± 3 kJ/mol for CO_2_ and CO methanation, independent of particle size (Figure S5d,e). The comparable surface kinetics and coverages of CH_4_ intermediates in CO_2_ and CO hydrogenation emphasize the shared structural requirements of these two reactions.

The changes in surface coverage as a function of particle size are also reflected in the reaction orders. With increasing particle size, the reaction order in H_2_ for the formation of CH_4_ decreases from 0.4 to 0.2, while the reaction order in CO_2_ increases from 0 to 0.1 (Figure 4b). In contrast to CH_4_ formation, the reaction order in CO_2_ for CO formation was approximately 0.8 for all particle sizes (Figure S5b). As a result, increasing CO_2_ pressure results in a higher CO pressure. Since CO is a well-known inhibitor of methanation,^33,90−92^ increasing CO pressure can affect CH_4_ formation rates. Small particles display a higher selectivity toward CO than large particles during CO_2_ hydrogenation (Figure 4c), and this selectivity results in a higher partial pressure of CO relative to the amount of CH_4_ intermediates. The stronger inhibition of CO on small particles explains the lower reaction order in CO_2_ for methanation when decreasing the particle size. Conversely, the inhibition of CH_4_ formation by CO results in a higher order in H_2_ because a higher H_2_ pressure enhances CO removal.^33,91^ Thus, even higher reaction orders in H_2_ can be expected for CO hydrogenation conditions. Indeed, a relatively high reaction order in H_2_ (0.6) and a low one in CO (−0.3) are obtained for all particle sizes during CO hydrogenation (Figure S5c). The trends in reaction orders illustrate the interplay between CO_2_ hydrogenation performance and particle size and reflect the changes in surface coverages and CO selectivity.

### 3.5 Mechanistic Relevance of Observed Surface Species

The above results demonstrate that the surface coverage of CH_4_ intermediates decreases with decreasing particle size. To establish the identity of these surface species, we applied operando DRIFTS, while simultaneously recording the catalyst performance by MS. The DRIFTS spectra recorded during CO_2_ hydrogenation are shown for the samples Ni2.7 (Figure 4d) and Ni5.9 (Figure 4e). All spectra contain stretching modes of adsorbed carbonyls (CO*) at 2060, 2030, 1925, and 1825 cm^–1^. The bands at 2060–2030 cm^–1^ can be assigned to linearly adsorbed carbonyl species, while those at 1925 and 1825 cm^–1^ correspond to, respectively, CO bridging between two Ni atoms and CO adsorbed on several Ni atoms.^93−97^ Notably, carbonyl band areas of the Ni2.7 sample are lower than those of the Ni5.9 sample, which coincides with the decrease in the coverage of intermediates as obtained from SSITKA (Figures S13 and 4a). Contrary to some recent reports,^25,98^ we did not observe major shifts in carbonyl band positions between the different particle sizes. From the present data, we infer that decreasing particle sizes lowers the coverage of carbonyl species but does not significantly affect the nature of the surface sites. Furthermore, the spectra in Figure 4d,e also contain bands at 2910 and 2860 cm^–1^ indicative of adsorbed formate species (HCOO*). The bands can be assigned to a combination of C–H stretching and bending vibrations.^99,100^ In contrast to the higher carbonyl coverage for the Ni5.9 sample, the intensity of the formate absorption band is lower for Ni5.9 and further decreases for larger particles of 12.2 nm (Figure S13). While the obtained IR spectra provide insights into the concentration and nature of the adsorbed species under steady-state conditions, it is not possible to assess the relevance of these species in the mechanism of CO_2_ hydrogenation. Therefore, combined DRIFTS–SSITKA experiments were performed to determine the reactivity of the surface species and their mechanistic relevance. This combination of tools can reveal key surface intermediates for various reactions.^92,101^

In DRIFTS–SSITKA experiments, we performed switches between ^12^CO_2_ + H_2_ and ^13^CO_2_ + H_2_ after reaching steady state in CO_2_ hydrogenation conditions. To amplify the differences in the residence times of surface species, we lowered the reaction temperature to 200 °C. From the identical Ne and CO_2_ transients obtained by MS, we can infer that the interaction of CO_2_ with the catalyst is weak (Figure S13a,b). Therefore, the gas-phase response of CO_2_ in the IR spectra can be used to account for the gas hold-up in the mean surface residence time calculations of the adsorbed species from DRIFTS data.

**Figure 5:**
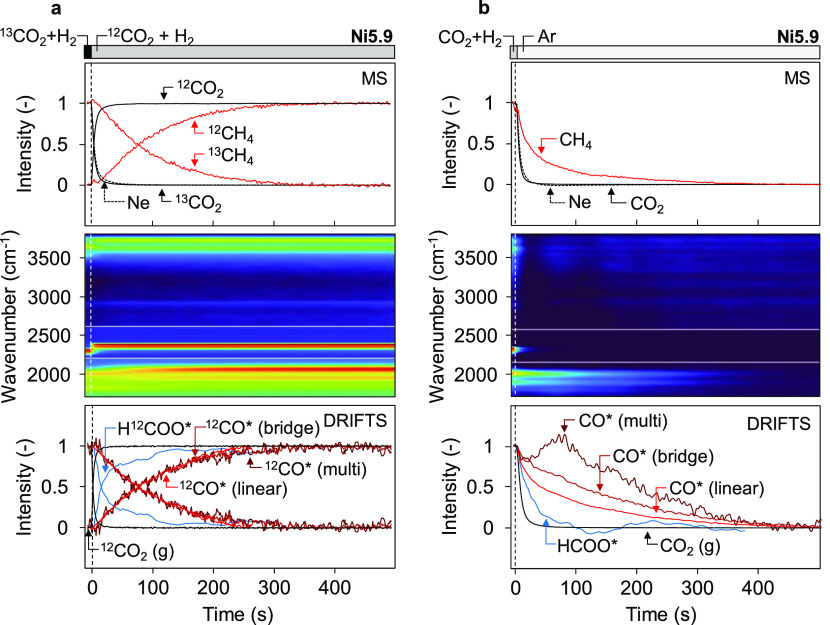
Surface dynamics of the adsorbed species. (a) Operando DRIFTS–SSITKA result of ^13^CO_2_ + H_2_ to ^12^CO_2_ + H_2_ switch during steady-state CO_2_ hydrogenation at 200 °C. Ne is added to the ^13^C-containing mixture to account for gas hold-up. The top panel displays the normalized intensities of Ne, ^12/13^CO_2_, and ^12/13^CH_4_ obtained by MS as a function of time. The middle panel shows DRIFTS spectra as a function of time. The bottom panel displays the normalized spectral response of the different gas/surface species determined by DRIFTS. (b) Desorption experiment of CO_2_ + H_2_ to Ar switch.

The DRITFS–SSITKA results for the Ni5.9 catalyst are shown in Figure 5a. In contrast to the fast replacement of Ne and CO_2_ observed by MS and IR, the CH_4_ transient is slower and resembles the transient of the carbonyl species. The mean surface residence time of CH_4_ intermediates determined from MS is 91 s, while the various carbonyl species from IR have a similar surface residence time of 93 s. Formate species have a substantially shorter surface residence time of 28 s. As shown in Figure S15, the τ_0_ values from SSITKA measurements in a regular fixed-bed reactor correspond well to the values obtained from DRIFTS–SSITKA. In addition, similar transients are obtained for the Ni2.7 sample (Figure S16). After the DRIFTS–SSITKA experiment, a switch to Ar was performed to determine the removal rates of the different surface species (Figure 5b). Formate species are rapidly removed from the surface with a rate similar to the rate observed in the SSITKA experiment. In contrast, clear differences are observed for the removal rates of carbonyl species. After the switch to Ar, linear carbonyl species are removed first, followed by bridged and, finally, multibonded species. For the multibonded species, the intensity gradually decreases after 100 s, which is slower than the surface residence time of these species during SSITKA switches.

Combining the observations of SSITKA and transient desorption experiments, we propose that during steady-state operation, the primary reaction pathway involves hydrogenation of strongly bonded carbonyls to CH_4_. The migration of the other carbonyl species to the sites that strongly bind CO and activate C–O bonds is faster than the formation of CH_4_.^102^ This results in identical surface residence times for the carbonyl species obtained during SSITKA switches. Notably, all carbonyl species are eventually converted into CH_4_ and, therefore, account for the CH_4_ intermediate coverage as determined from SSITKA experiments.

**Figure 6:**
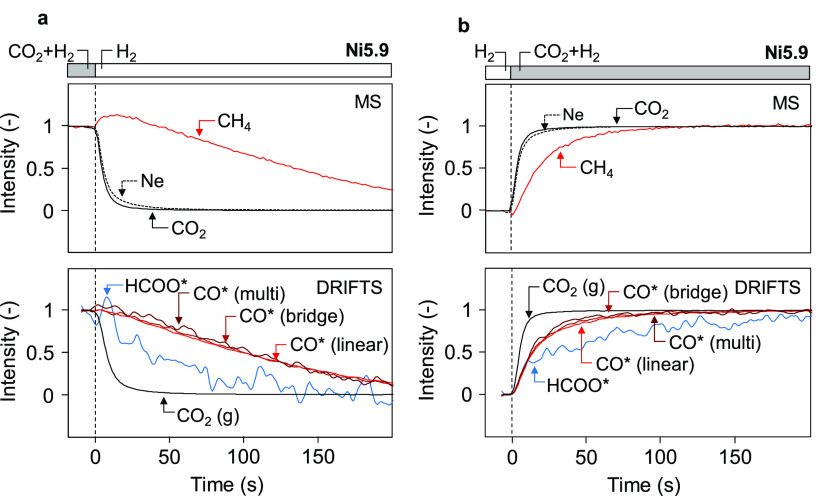
Response of adsorbed species during switches between CO_2_ + H_2_ and H_2_. Transients of gas and surface species from MS and DRIFTS during switch from CO_2_ + H_2_ to H_2_ (a) and from H_2_ to CO_2_ + H_2_ (b) of the Ni5.9 sample (200 °C, 50 mL min^–1^, 5 kPa CO_2_, 20 kPa H_2_ in Ar).

The relevance of formate species in the CO_2_ hydrogenation mechanism is still subject to debate.^92,103−105^ Although DFT calculations have reported lower barriers for direct CO_2_ dissociation on Ni, Rh, and some other transition metals,^106−108^ several studies suggest that H-assisted CO_2_ activation via decomposition of formate intermediates to CO is the main reaction pathway for the RWGS.^92,109^ From the present data, the similar surface residence times of CO intermediates from SSITKA (Figure S15) and formate species from DRIFTS–SSITKA point at a link between formate species and CO formation. Further insights into the role of formate species for CO formation were obtained by following the transient behavior of the surface species during switches between H_2_ and CO_2_ + H_2_. As shown in Figure 6a, formate species are removed faster than carbonyl species after a switch from CO_2_ + H_2_ to H_2_. The carbonyl removal rates are comparable to the rates obtained during the DRIFTS–SSITKA experiment since in both cases, the carbonyl transients reflect the conversion of carbonyls to methane. Switching from H_2_ to CO_2_ + H_2_ results in the opposite behavior, where the build-up of carbonyl species is faster than that of formate species (Figure 6b). Here, the carbonyl coverage is likely governed by the adsorption of CO molecules derived from CO_2_ conversion. The fast increase in CH_4_ formation rates might then be linked to the relatively high hydrogen coverage when CO_2_ is added to the feed. Similar results were obtained for different particle sizes (Figure S17). Additional in situ IR experiments demonstrate that exposing an empty Ni surface to CO_2_ without H_2_ present readily yields carbonyl species (Figure S18). As expected, formate bands were only observed after the introduction of H_2_. Moreover, the coverage of formate species decreases when increasing particle size (Figure 4d,e) and seems proportional to the length of the Ni–support perimeter (Figure S13d). Therefore, we suggest that the formate species, likely located at the interface between Ni nanoparticles and the support,^99,110,111^ have a minor contribution to the overall CO_2_ hydrogenation rates. Since the surface-specific CO_2_ conversion rates are weakly dependent on particle size, CO is proposed to be primarily formed by the direct dissociation of CO_2_ on the Ni surface. This is in line with previous theoretical calculations, where direct dissociation of CO_2_ was found to be the dominant pathways for the formation of CO.^107,108^ The (reversible) decomposition of formate species to CO could contribute to an increased surface residence time of intermediates leading to gaseous CO, which provides an explanation for the similar surface residence time values observed for SSITKA and DRIFTS–SSITKA measurements.

### 3.6 General Discussion

The obtained results provide insights into the structural requirements of reaction pathways in CO_2_ hydrogenation. First, kinetic measurements and operando DRIFTS demonstrate the sequential nature of CO_2_ methanation, where RWGS precedes the CO methanation reaction. Second, the manner in which the Ni particle size affects CO_2_ hydrogenation performance shows that the RWGS and CO methanation reactions display different structure sensitivity. Surface-specific CH_4_ formation rates decline sharply when decreasing the particle size below 5 nm, while the RWGS rate is unaffected. Third, the surface residence times derived from SSITKA measurements and the apparent activation energies of CO and CH_4_ (Figure S3d,e) emphasize the invariant intrinsic turnover rates of the two reactions as a function of particle size. With this, we elucidate that the surface sites responsible for the RWGS and CO methanation reactions are different.

Previous studies have addressed the structure sensitivity of the RWGS reaction, but its exact structural requirements are still subject of debate.^22,109,112−114^ Nevertheless, the RWGS reaction has been reported to occur even on single atoms, clusters, and (bimetallic) nanoparticles.^22,113−117^ The weak dependence of RWGS on the catalyst structure is in stark contrast with CO methanation, which is known to require specific step edges for the dissociation of CO.^118−121^ Thus, we suggest that CO is readily formed from CO_2_ dissociation on most Ni surface sites. The observed formate species are proposed to have a minor contribution to the overall CO formation rates. The produced CO molecules from CO_2_ dissociation adsorb on the surface in linear, bridged, and multibonded adsorption configurations. Since CH_4_ formation rates are governed by the slow conversion of carbonyl species on step edge sites, the migration of carbonyl species to these sites is much faster than their conversion to CH_4_. This results in equivalent surface residence times of the different carbonyl species observed during DRIFTS–SSITKA experiments.

In the literature, the particle size effects in CO_2_ or CO methanation have been attributed to various phenomena such as oxidation, restructuring, and poisoning (further details in Note S7). From thermodynamic calculations, small metal nanoparticles are more likely to oxidize under high H_2_O/H_2_ or CO_2_/CO ratios, particularly at high temperatures.^122,123^ However, at the conditions used in this study, operando XANES experiments evidence that oxidation during CO_2_ hydrogenation is negligible, even for small Ni particles. While surface oxidation by CO_2_ or H_2_O can be expected at elevated temperatures and pressures,^25,39^ we argue that it is not the main reason for the particle size effect observed here. With regard to restructuring, no significant variations on coordination numbers, lattice parameters, and strain were observed when exposing the catalysts to CO_2_ hydrogenation conditions. Accumulation of adsorbed CO or CH_x_ species for smaller particles, often linked to site blocking or poisoning,^6,54,56^ was absent as shown by DRIFTS experiments.

As discussed above, we found that the nature of the active sites responsible for CO and CH_4_ formation does not change with the particle size. Instead, the number of surface sites responsible for the hydrogenation of strongly bonded carbonyl species to CH_4_ decreases with decreasing particle size. In the literature, monoatomic steps are thought to be essential for the activation of CO,^12,121,124,125^ and relatively large terrace overlayers are required to stabilize these sites.^4,81,126^ Theoretical simulations have indicated that the number of step sites and the associated terrace overlayer size decreases with decreasing particle size.^4,81^ Therefore, the decrease in methanation activity for smaller particles is typically attributed to the change in the number of these reactive surface sites with size. In some cases, it has also been speculated that the interior atoms in small nanoparticles may not yet have a structure that corresponds to the most stable bulk structure.^4,74,81,127,128^ In our study, we observed an increased stacking fault probability by XRD for smaller particles. A strong surface strain is considered to be the driving force for the distortion of crystallite bulk structures, and it typically increases with decreasing particle size.^71−80^ Previous studies have pointed out that the relative stability of particular surface geometries can be affected by differences in bulk structure.^81−88^ Given these findings, it is worthwhile to further investigate how bulk and surface defects are correlated and how they can be used to better understand the strong structure sensitivity of metal catalysis by nanoparticles in the 1–10 nm range.

## 4 Conclusions

In this work, operando spectroscopic and kinetic experiments provided mechanistic insights into the structure sensitivity of key reaction steps in CO_2_ hydrogenation on Ni catalysts. CO_2_ hydrogenation was found to proceed via the RWGS reaction, followed by CO methanation. The observed particle size effects revealed different Ni surface sites to catalyze these two reactions. The conversion of CO_2_ to CO is unaffected by changes in particle size, while CO methanation rates sharply decline when decreasing particle sizes below 5 nm. Notably, the intrinsic activity and active sites of both reaction pathways do not depend on particle size. We did not observe any significant CO_2_- or CO-induced oxidation, restructuring, or poisoning of Ni nanoparticles, which could explain the observed particle size effect. Instead, we linked the decreased methanation rates to a lower density of reactive surface sites on the smaller particles. The change in surface structure was also reflected in the surface coverages, where the coverage of intermediates linked to CH_4_ formation declined when decreasing the particle size. Carbonyl species were identified as intermediates for the formation of CH_4_, while formate species contribute little to the overall CO_2_ hydrogenation rate. Combining operando characterization with detailed kinetic analysis allowed us to follow key reaction sequences on complex heterogeneous surfaces. The results of this work emphasize the mechanistic connection between CO_2_ and CO hydrogenation, which require the same surface sites for the conversion of *CO intermediates. Consequently, the observed structure–performance relationships can be valuable for the design of more active and selective catalysts for CO_2_ hydrogenation and Fischer–Tropsch synthesis.

## Supporting Information Available

Additional experimental details and results, testing for heat and mass transport effects, and literature overview on particle size effects for CO_2_ and CO hydrogenation (PDF)
