# Supplementary material for: Structure Sensitivity of CO2 Hydrogenation on Ni Revisited
Source: J Am Chem Soc. 2023 Sep 7;145(37):20289–301. doi: 10.1021/jacs.3c04284 (PMC10515628; doi:10.1021/jacs.3c04284)
Supplement: Supplementary file 1 — ja3c04284_si_001.pdf [file ja3c04284_si_001.pdf]

Structure sensitivity of CO_2 hydrogenation on Ni revisited

Jérôme F.M. Simons, Ton J. de Heer, Rim C.J. van de Poll, Valery Muravev, Nikolay Kosinov, Emiel J.M. Hensen*

Laboratory of Inorganic Materials and Catalysis, Department of Chemical Engineering and Chemistry, Eindhoven University of Technology, P.O. Box 513, 5600 MB Eindhoven, The Netherlands

Supplementary information

Characterization

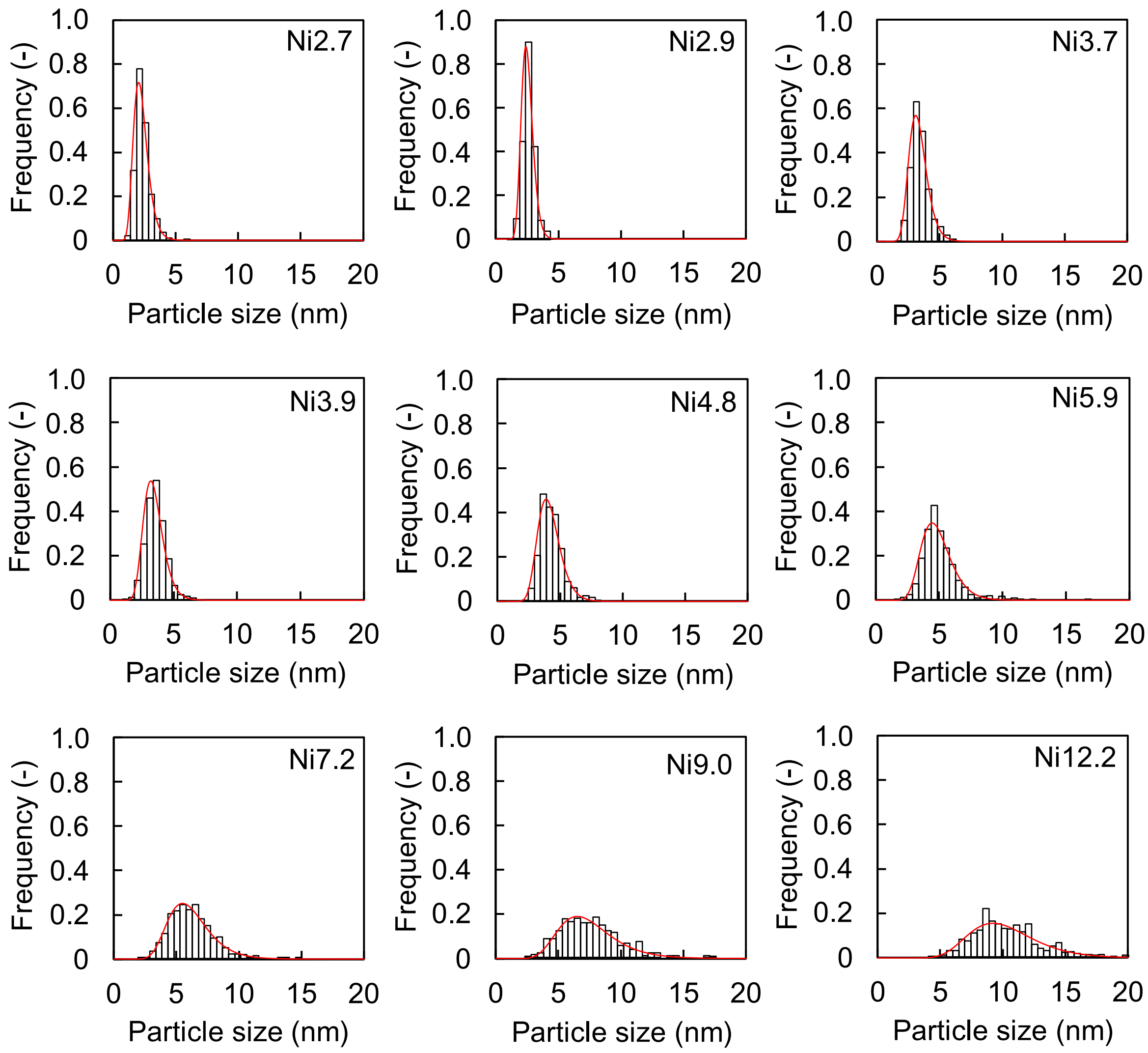

Figure S1. Particle size distributions (N = 500) from HAADF-STEM of Ni/SiO_2 catalysts after H_2-pretreatment.

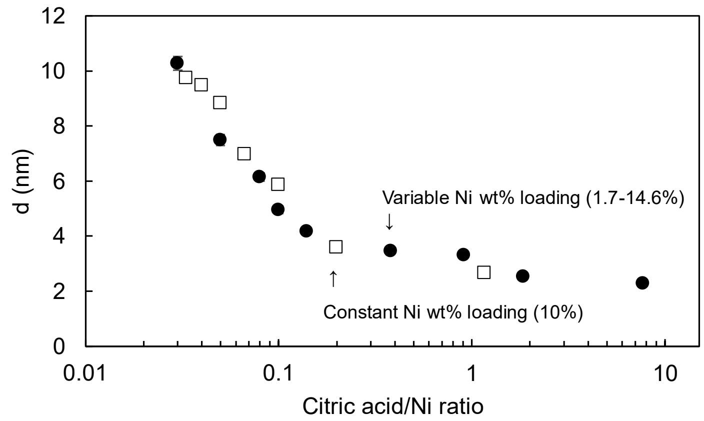

Figure S2. Effect of citric acid concentration and Ni weight loading on Ni particle size. Citric acid/Ni ratio versus Ni particle size, determined after H_2 pretreatment, of samples containing variable Ni wt% loading (solid circles) and constant Ni wt% loading of 10% (open squares).

Sample	Ni content (wt%)[a]	Citric acid/ Ni ratio (-)[b]	$d_{n,STEM}$ (nm)[c]	$\sigma_{n,STEM}$ (nm)[d]	$d_{a,STEM}$ (nm)[c]	d_{H_2} (nm)[e]	d_{XRD} (nm)[f]
Ni2.7	1.7 ± 0.0	7.69	2.3 ± 0.1	0.6 (27%)	2.7 ± 0.1	2.7 ± 0.2	
Ni2.9	2.4 ± 0.0	1.85	2.5 ± 0.0	0.5 (19%)	2.8 ± 0.1	2.6 ± 0.1	
Ni3.7	3.3 ± 0.1	0.91	3.3 ± 0.1	0.7 (23%)	3.7 ± 0.1	2.9 ± 0.2	3.8 ± 0.3
Ni3.9	4.8 ± 0.1	0.38	3.5 ± 0.1	0.8 (23%)	3.9 ± 0.1	3.0 ± 0.2	3.8 ± 0.3
Ni4.8	9.4 ± 0.0	0.14	4.2 ± 0.1	0.9 (22%)	4.8 ± 0.1	5.3 ± 0.3	5.3 ± 0.4
Ni5.9	7.9 ± 0.3	0.10	5.0 ± 0.1	1.5 (31%)	5.9 ± 0.2	5.1 ± 0.3	6.5 ± 0.4
Ni7.2	10.0 ± 0.2	0.08	6.1 ± 0.2	1.7 (28%)	7.2 ± 0.2	7.3 ± 0.4	7.5 ± 0.4
Ni9.0	11.9 ± 0.3	0.05	7.5 ± 0.2	2.3 (31%)	9.0 ± 0.2	10.2 ± 0.6	9.1 ± 0.3
Ni12.2	14.6 ± 0.4	0.03	10.3 ± 0.2	2.8 (28%)	12.2 ± 0.3	14.2 ± 0.8	11.0 ± 0.5

Table S1. Characterization of Ni/SiO_2 catalysts after reduction, including 95% confidence intervals. [a]Ni weight content determined from ICP-OES, with ± 95% confidence interval values. [b]Nominal citric acid to Ni precursor molar ratio during incipient-wetness impregnation. [c]Mean area-weighted particle size from HAADF-STEM of 500 particles after reduction. [d]Particle size after reduction from H_2-chemisorption measurements at 35°C. [e]H_2-uptake from H_2-chemisorption measurements. [f]Particle size after reduction from XRD using the Scherrer equation.

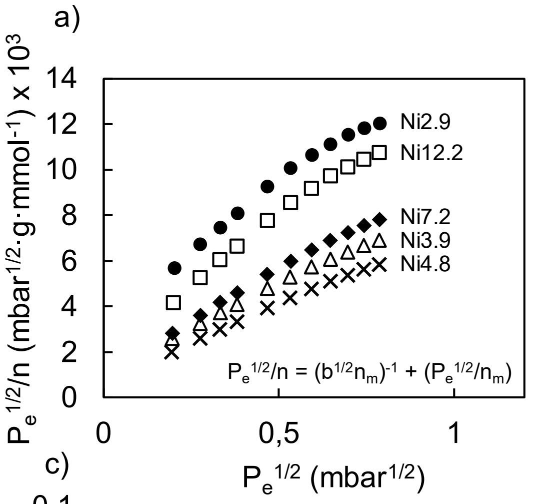

b)

	H_2 uptake ($\mu mol_{H2}\ g_{cat}^{-1}$)
Ni2.7	54.6 ± 3.1
Ni2.9	81.3 ± 4.6
Ni3.7	99.0 ± 5.6
Ni3.9	136.1 ± 7.7
Ni4.8	153.0 ± 8.7
Ni5.9	134.0 ± 7.6
Ni7.2	117.6 ± 6.7
Ni9.0	100.4 ± 5.7
Ni12.2	88.5 ± 5.0

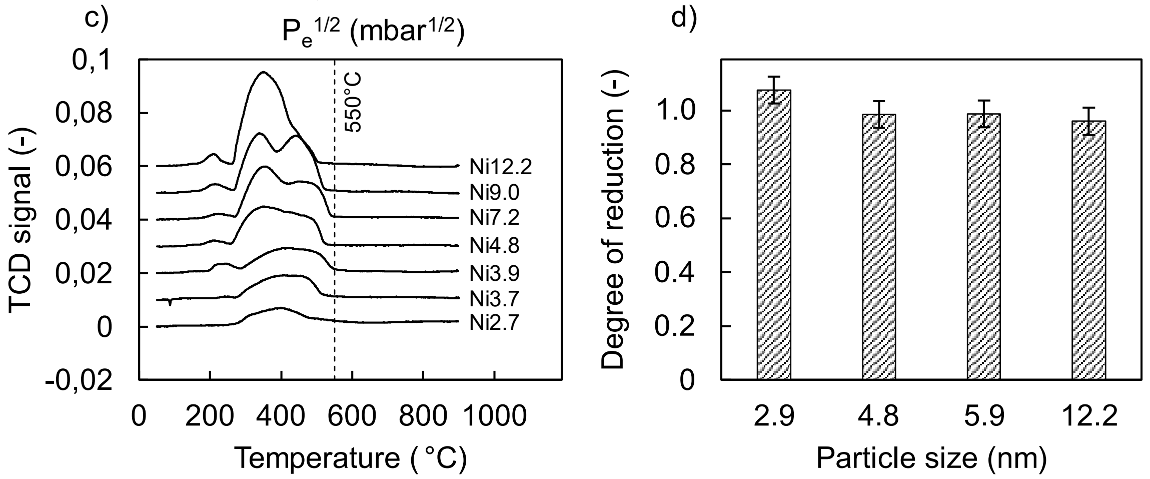

Figure S3. a) Example of H_2-chemisorption results plotted according to the linearized form of the Langmuir equation for dissociative adsorption[1,2] . The reciprocal of the slope of the initial linear part gives the monolayer amount n_m. No adsorption of H_2 on the blank support was observed. b) H_2 uptake from H_2-chemisorption, including the ± 95% confidence intervals. c) H_2-TPR of various Ni/SiO_2 samples. As is indicated by the dotted line, no more H_2 is consumed after reaching 550°C. d) O_2-titration after H_2 pretreatment at 550°C (5°C/min, 4h dwell). Degree of reduction is calculated with the weight loading from ICP-OES and under the assumption of 2Ni + O_2 → 2NiO.

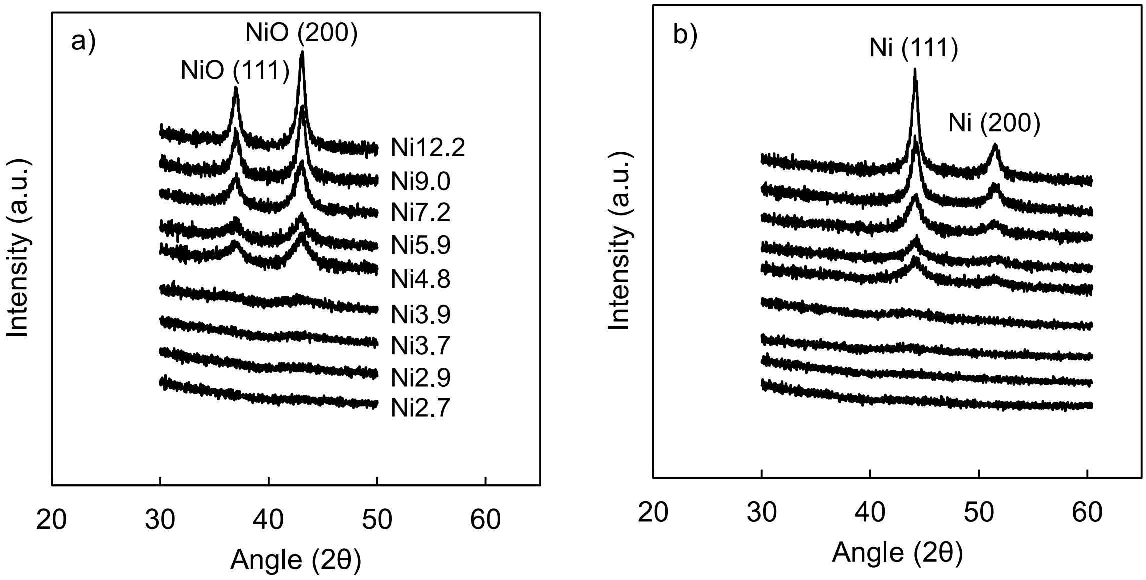

Figure S4. XRD patterns of the a) fresh and b) reduced Ni/SiO_2 catalyst. The reduced samples were pretreated at 550°C and 10% H_2/He for 4 hours and passivated in 2% O_2/He at room temperature before XRD analysis.

**Sample**	**d_{NiO} (nm)**	**d_{Ni} (nm)**
Ni2.7	n.a.	n.a.
Ni2.8	n.a.	n.a.
Ni3.7	4.2 ± 0.5	3.8 ± 0.3
Ni3.9	4.6 ± 0.4	3.8 ± 0.3
Ni4.8	5.5 ± 0.3	5.3 ± 0.4
Ni5.9	5.6 ± 0.3	6.5 ± 0.3
Ni7.2	7.4 ± 0.3	7.5 ± 0.4
Ni9.0	9.2 ± 0.3	9.1 ± 0.3
Ni12.2	10.8 ± 0.5	11.0 ± 0.5

Table S2. Crystallite sizes from XRD, including ± 95 % confidence interval values. Due to the low intensity of both NiO and Ni peaks, it was not possible to determine the crystallite sizes of Ni2.7 and Ni2.8. Crystallite sizes were determined using the Scherrer equation and integral breadth β_i from the NiO(200) or Ni(111) peaks.

Catalyst performance

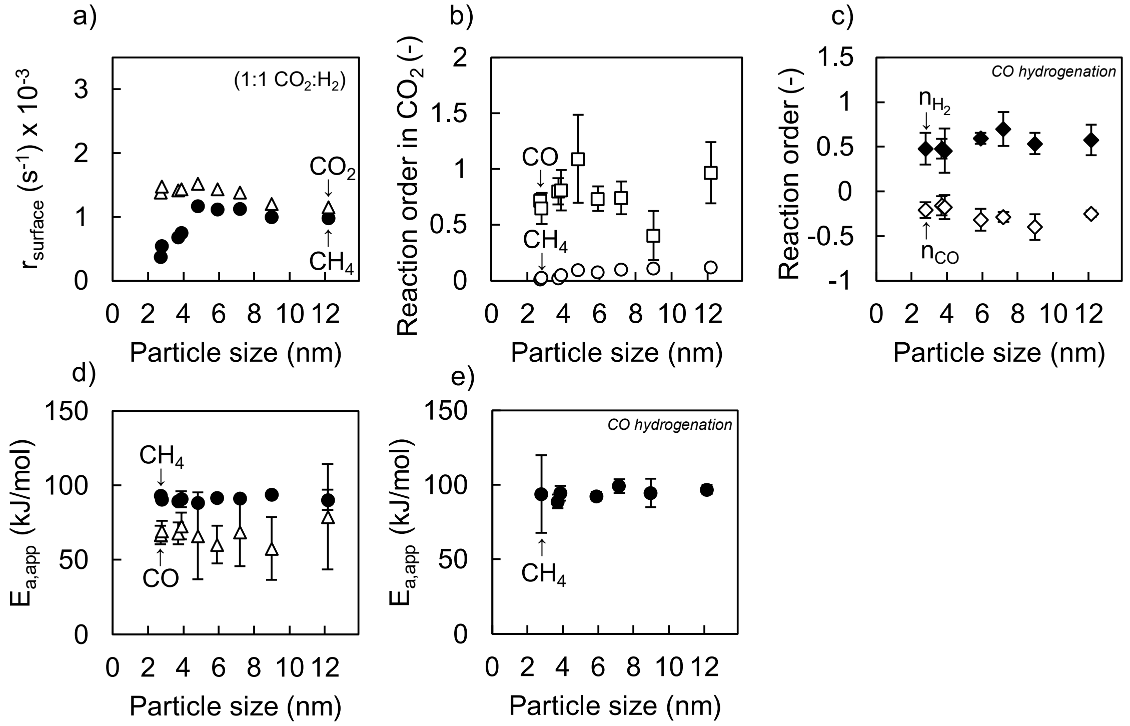

Figure S5. Activity of Ni/SiO_2 catalysts at 220 °C. a) Surface-specific rates of CO_2 (open triangles) and CH_4 (solid circles) versus particle size during CO_2 hydrogenation (50 mL/min 5 vol% CO_2 and 5 vol% H_2 in Ar). b) Reaction order in CO_2 for CO (open squares) and CH_4 (open circles) formation during CO_2 hydrogenation. c) Reaction order in H_2 (solid diamonds) and CO (open diamond) for CH_4 formation during CO hydrogenation (50 mL/min 2 vol% CO and 20 vol% H_2 in Ar). d) Apparent activation energy for CH_4 (solid circles) and CO (open triangles) formation during CO_2 hydrogenation. $E_{a,app}$ is determined from activity results between 216 and 224 °C, with intervals of 2 °C. e) Apparent activation energy for CH_4 formation during CO hydrogenation.

Steady-state isotopic transient kinetic analysis

Note S1. Steady-state isotopic transient kinetic analysis.

Steady-state isotopic transient kinetic analysis (SSITKA) was used to determine the surface-residence time and coverage of the intermediates[3]. At a certain moment in time during steady-state operation, we rapidly replace $^{12}CO_2$ with $^{13}CO_2$ (99% ^{13}C, Eurisotop) and add Ne as tracer. By following the transient response of the products relative to the Ne tracer, we are able to determine the surface-residence time of the product intermediates. The mean surface-residence time $\bar{\tau}^P$ was calculated by integrating the time response of $^{12}C/^{13}C$ replacement in CH_4 and CO according to:

$$\bar{\tau}^P = \int_0^{\infty} (F^P(t) - F^{Ne}(t))dt \tag{1}$$

Where F^P the normalized transient response of the product P and F^{Ne} the normalized transient response of the tracer Ne. From the mean surface-residence time, the amount of intermediates and their fractional coverage are determined by:

$$\bar{N}^P = \bar{\tau}^P \cdot r^P \tag{2}$$

$$\bar{\theta}_c{}^P = \frac{\bar{N}^P}{\bar{N}_c} = \bar{\tau}^P \cdot \overline{TOF}^P \tag{3}$$

Where $\bar{N}^P$ is the total number of surface intermediates, r^P the steady-state reaction rate, $\bar{\theta}_c{}^P$ the fractional surface coverage and $\bar{N}_c$ the Ni surface area, determined by H_2-chemisorption. The transients of $^{12}CH_4$ (m/z = 15), $^{13}CH_4$ (m/z = 17), ^{12}CO (m/z = 28), ^{13}CO (m/z = 29), $^{12}CO_2$ (m/z = 44) and $^{13}CO_2$ (m/z = 45) were monitored by an online quadrupole mass spectrometer (ESS, GeneSys Evolution).

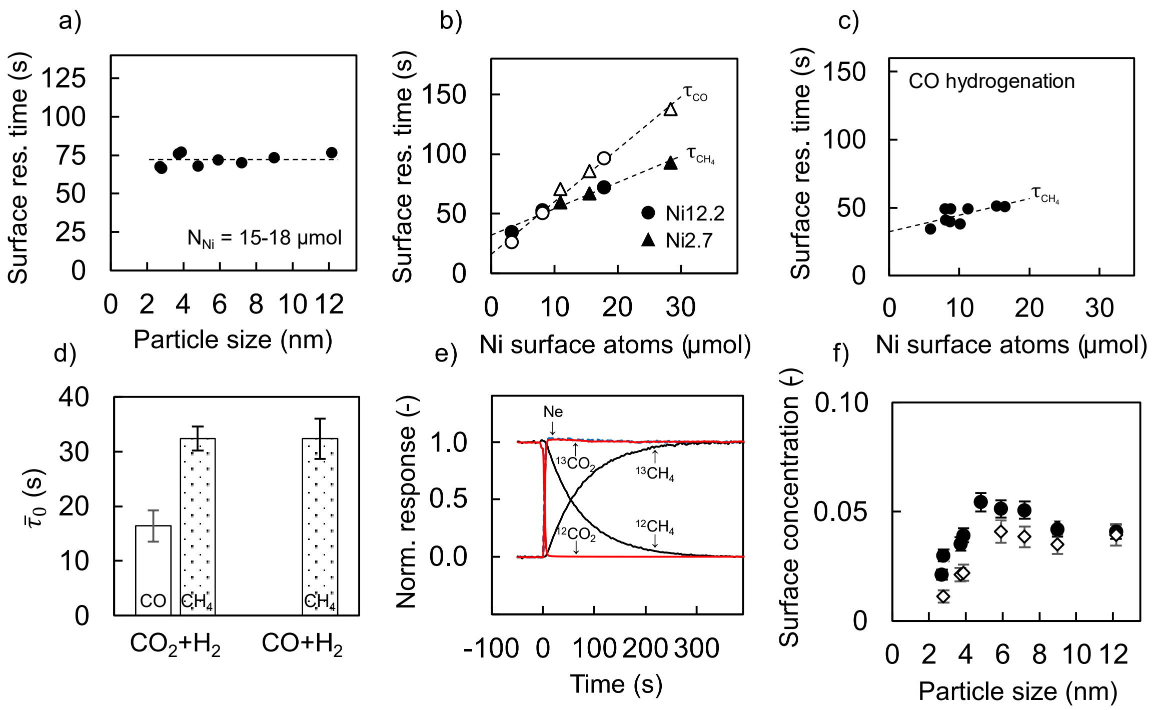

Figure S6. Surface residence times and surface concentration during CO_2 and CO hydrogenation at 220°C from SSITKA. a) Uncorrected surface residence time of CH_4 intermediates of different particle sizes at constant amount of surface Ni loaded in the reactor, b) Surface residence time of CH_4 (solid symbols) and CO (open symbols) intermediates for Ni2.7 (circles) and Ni12.2 (triangles) versus amount of surface Ni during CO_2 hydrogenation. Note that the surface residence times of CO have been corrected for the hydrogenation of readsorbed CO to CH_4 (see Supplementary Note S2). c) Surface residence time of CH_4 precursors of various Ni nanoparticles versus amount of surface Ni during CO hydrogenation, d) Readsorption corrected surface residence times of CH_4 and CO precursors, e) Example of SSITKA transient during CO_2 hydrogenation of Ni5.9 sample, f) Surface concentration of CH_4 precursors during hydrogenation of CO_2 (solid circles) and CO (open diamonds).

Note S2. Correction of CO surface residence times from SSITKA measurements. The measured surface residence time of CO intermediates only accounts for CO desorbing from the surface and leaving the reactor in the effluent stream. Here, the readsorption and hydrogenation of CO throughout the reactor is not reflected by the obtained surface residence time. Only the CO intermediates that desorb and leave the reactor in the effluent flow are responsible for the observed surface residence time. This results in a non-linear behavior of the surface residence time as a function of the amount of exposed Ni atoms (shown in the left figure below), since varying the amount of Ni surface atoms affects the probability of re-adsorption and hydrogenation of CO. We used the SSITKA results of the set of catalyst samples to estimate the correlation between the CO surface residence time and the CH_4 selectivity (shown in the right figure). With this estimation, the corrected CO surface residence time could be obtained.

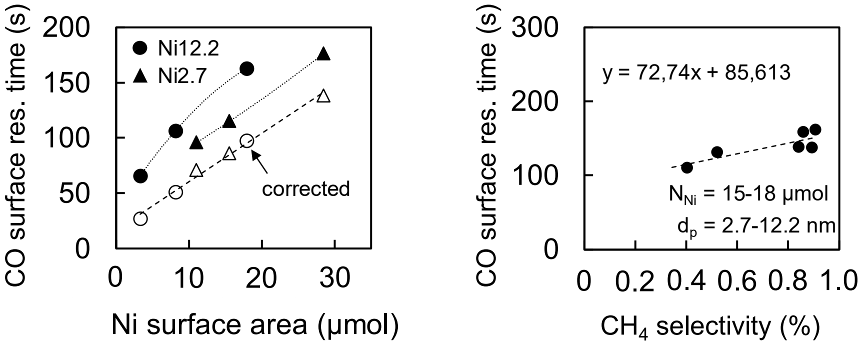

X-ray absorption spectroscopy and X-ray diffraction

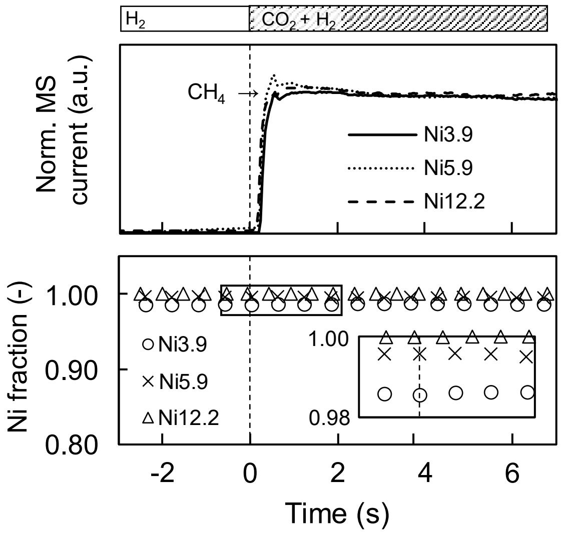

Figure S7. Linear combination fitting analysis of operando XANES data during a switch from H_2 to CO_2 + H_2 (50 ml/min, 5 vol% CO_2 and 20 vol% H_2 in Ar, 220 °C). The normalized CH_4 concentration (m/z = 15) from MS is shown in the top figure. The bottom figure includes the $Ni^0/(Ni^{2+} + Ni^0)$ fraction obtained from linear combination fitting. Since the coordination numbers of the NiO and Ni nanoparticles are lower than their bulk counterparts (Table S3), the XANES features of the nanoparticles differ from those of bulk NiO and Ni. Therefore, we used the spectra of the NiO particles prior to the H_2 pretreatment step and the spectra of the Ni nanoparticles after the pretreatment as standards for the linear combination fitting.

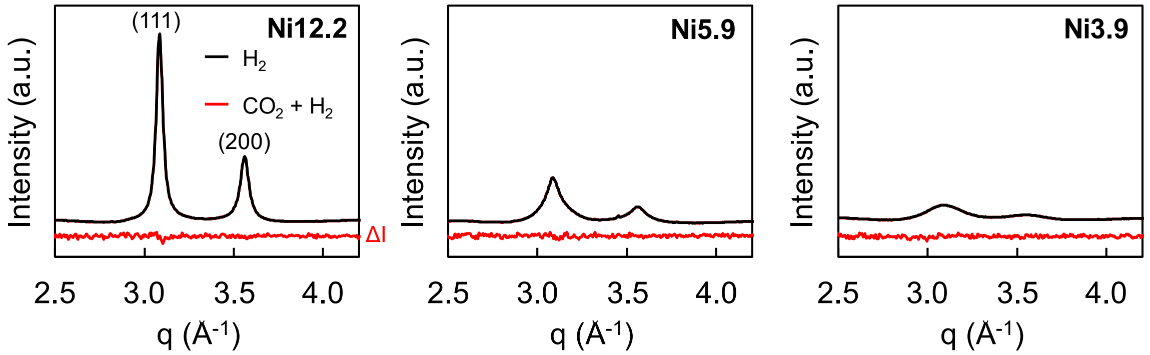

Figure S8. operando XRD results of the Ni12.2 (left panel), Ni5.9 (middle panel) and Ni3.9 (right panel) samples exposed to H_2 (solid black line) and CO_2 + H_2 (solid red line). The difference between the two XRD results (ΔI) is displayed below the diffractograms.

	Ni foil	Ni12.2	Ni5.9	Ni3.9
ΔE_0 (eV)	-3.9 ± 0.5	-3.7 ± 0.5	-3.5 ± 0.4	-3.1 ± 0.4
σ_{SS1}^2 (Å^2)	0.0059 ± 0.0004	0.0064 ± 0.0003	0.0072 ± 0.0003	0.0078 ± 0.0002
C_3 (Å^3)	0.00017 ± 0.00009	0.00020 ± 0.00008	0.00025 ± 0.00007	0.00039 ± 0.00007
σ_{Other}^2 (Å^2)	0.0102 ± 0.0010	0.0111 ± 0.0008	0.0114 ± 0.0009	0.0122 ± 0.0010
N_{SS1} (-)	11.3 ± 0.6	9.2 ± 0.4	8.9 ± 0.3	8.4 ± 0.3
r_{SS1} (Å)	2.497 ± 0.008	2.500 ± 0.006	2.504 ± 0.005	2.5112 ± 0.005
N_{SS2} (-)	6.4 ± 1.2	5.5 ± 0.9	4.1 ± 0.7	3.6 ± 0.7
r_{SS2} (Å)	3.535 ± 0.005	3.537 ± 0.004	3.538 ± 0.004	3.541 ± 0.004
N_{SS3} (-)	29.6 ± 3.3	23.1 ± 2.2	19.1 ± 1.9	15.9 ± 1.6
r_{SS3} (Å)	4.329 ± 0.006	4.332 ± 0.005	4.333 ± 0.005	4.336 ± 0.004
N_{SS4} (-)	14.8 ± 1.9	12.2 ± 1.4	9.7 ± 1.1	7.4 ± 0.9
r_{SS4} (Å)	4.999 ± 0.007	5.002 ± 0.006	5.004 ± 0.005	5.007 ± 0.005
N_{TR1} (-)	46.0 ± 11.6	38.1 ± 8.1	36.5 ± 6.3	32.4 ± 4.7
R-factor (%)	0.3	0.2	0.2	0.1

Table S3. Multiple-scattering EXAFS analysis results. The EXAFS data was acquired at room temperature in 10 vol% H_2 in Ar, after the reduction of the Ni/SiO_2 catalysts at 550 °C. The k-range used for the fitting was from 2.5 to 12.3 and the R-range from 1.7 to 5.1. A S_0^2 value of 0.92 was obtained by fitting the Ni foil reference. The error indicates the 95% confidence interval of the fitted parameters obtained from the Artemis software. Single scattering (SS), triangular scattering (TR) and collinear focusing double/triple-scattering paths from the 1st up to the 4th shell were included in the fitting. Coordination numbers (N) were varied independently for most scattering paths, with exception to the TR paths (constrained to be the same as the N of the first triangular scattering path) and the double/triple scattering paths (constrained to the N of the single scattering path with similar path length).[4,5] Since the distance dependence of the Debye-Waller factor σ^2 is generally weak for R >3 Å [6,7], one σ^2 parameter was used for the first SS path (SS1) and one for the other scattering paths. In addition, the third cumulant (C_3) was included for the pair distribution of the SS1 path. Finally, the nearest neighbour distances of the scattering paths, excluding SS1, were constrained by an isotropic lattice expansion parameter, using the relationship $R_i = (\epsilon + 1)R_{i,eff}$, where ϵ is the expansion factor and $R_{i,eff}$ is the path length predicted by fcc space group rules.

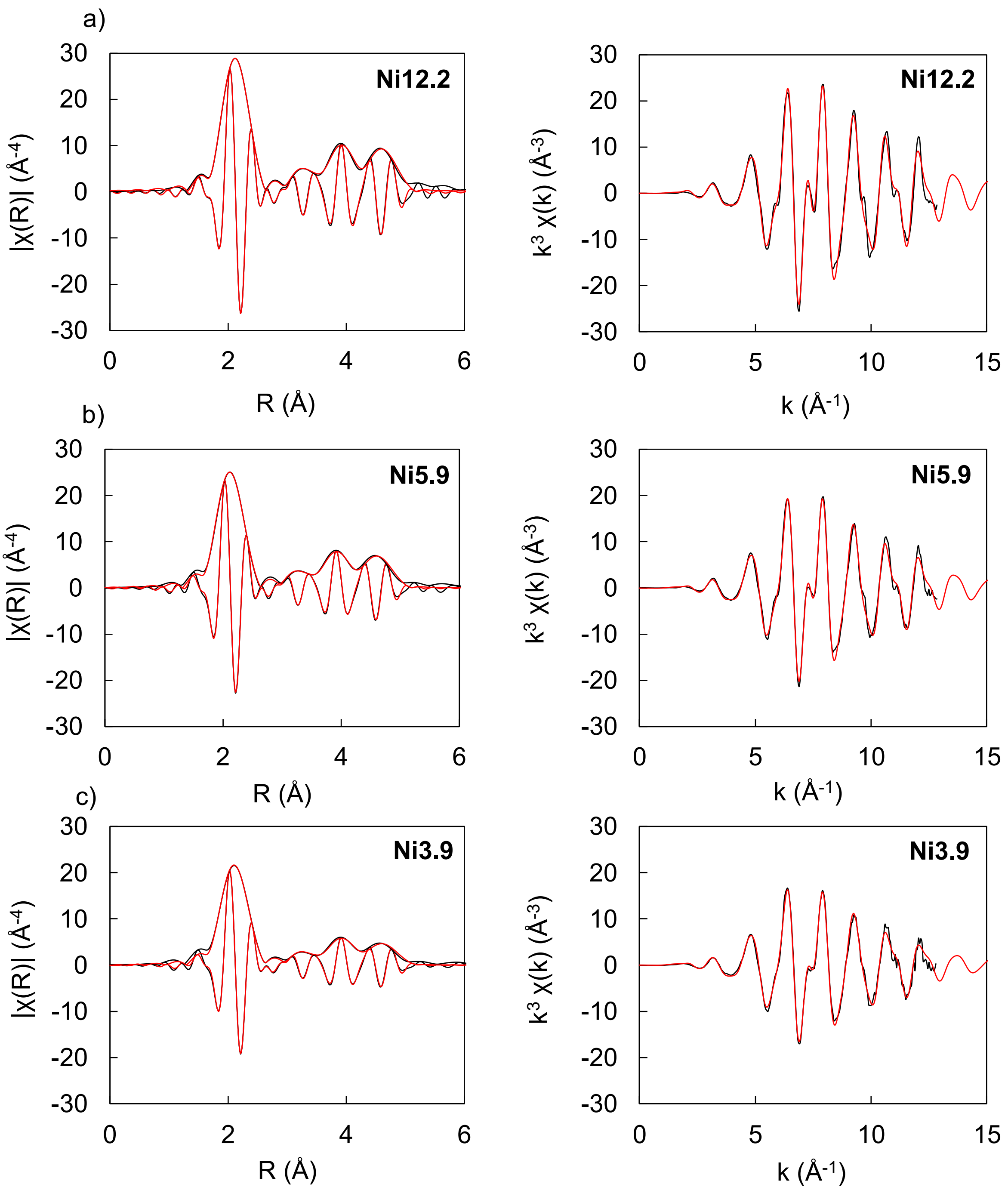

Figure S9. R-space (left figures), including the magnitude and real part of the Fourier transform, and k-space (right figures) of EXAFS data obtained at room temperature (after H_2 pretreatment) for the Ni12.2 (a), Ni5.9 (b) and Ni3.9 (c) samples. The black line represents the experimental data and the red line the fit. Details of the fitting results are shown in Table S3.

Note S3. Deformation fault probability.

Whole powder pattern modelling (WPPM) was used for the microstructural analysis of the Ni nanoparticles. The least squares minimisation software PM2K[8], which is based on a convolutive Fourier approach, was applied to fit the diffractograms obtained from XRD measurements at 220 °C (50 ml/min, 10 kPa H_2 in Ar). To model the peak profiles, the lattice parameter, domain size distribution and deformation fault probability are refined. The results of the WPPM method for the different Ni catalysts can be found in Figure S10 and S11.

Alternatively, manual analysis of individual peaks was used to estimate de deformation fault probability. According to Warren[9], the peak position shift as a function of deformation fault probability α is obtained from:

$$\Delta(2\theta)\ (°) = \frac{90\sqrt{3}\tan(\theta_0)}{\pi^2 h_0^2 m^{hkl}} \alpha \sum \pm L_0 \qquad (1)$$

where $\Delta(2\theta)$ is the peak displacement, θ_0 the ideal peak position of the reflection, h_0^2 is the sum of the squared Miller indices, m^{hkl} the number of affected and unaffected planes for a given fault plane, and L_0 the inner product of vectors normal to the fault plane and the reflecting plane. Since the peak displacement due to deformation faults is typically small, other sources of peak displacement, for example incorrect sample positioning or changes in the cell dimension, can affect the results. Therefore, reflection pairs with displacement in the opposite direction are used to calculate α, with the spacing between the (111) and (200) peaks, $\Delta(2\theta_{222} - 2\theta_{111})$, being most commonly used. However, the (111) peak displacement can deviate from the linear relation with α, which could lead to errors in the calculations.[10,11] Therefore, we included the peak displacement of the (200)–(220), (331)–(200) and (222)–(400) pairs to calculate the average α values.

a)

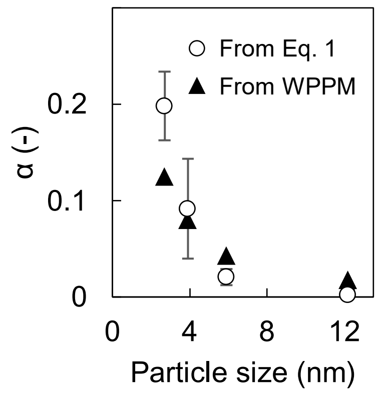

b)

	a (Å)	μ*	σ*	α (%)	R_{wp} (%)
**Ni12.2**	3.528 (± 5·10^{-7})	1.75 (± 3·10^{-4})	0.54 (± 9·10^{-5})	1.8 (± 4·10^{-4})	7.8
**Ni5.9**	3.526 (± 2·10^{-7})	1.14 (± 4·10^{-4})	0.43 (± 1·10^{-4})	4.3 (± 1·10^{-3})	8.2
**Ni3.9**	3.529 (± 6·10^{-7})	0.75 (± 4·10^{-4})	0.33 (± 2·10^{-4})	8.0 (± 3·10^{-3})	9.4
**Ni2.7**	3.531 (± 1·10^{-6})	0.54 (± 8·10^{-4})	0.37 (± 3·10^{-4})	12.5 (± 6·10^{-3})	13.5

Figure S10. a) Deformation fault probability α versus particle size as determined from the analysis of the peak positions using equation 1 (open circles) and from WPPM (solid squares). b) Table with the fitting results from the WPPM modelling. *) μ and σ are the mean and variance (squared) of the lognormal domain-size distribution.

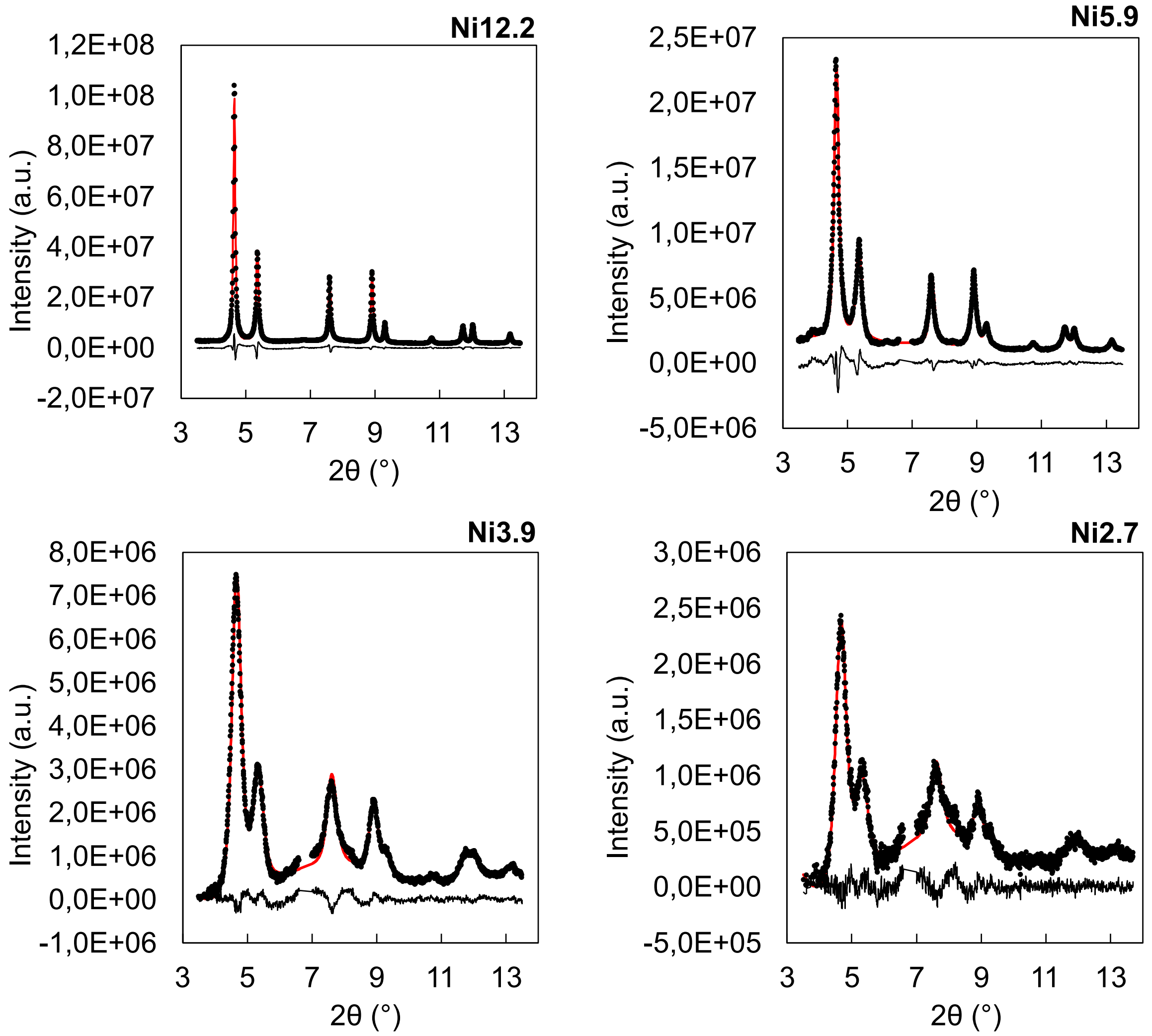

Figure S11. Whole powder pattern modelling results, including the data from XRD (black dots) and WPPM fitting results (red line). The residual between the experimental data and fitting is shown below the profiles.

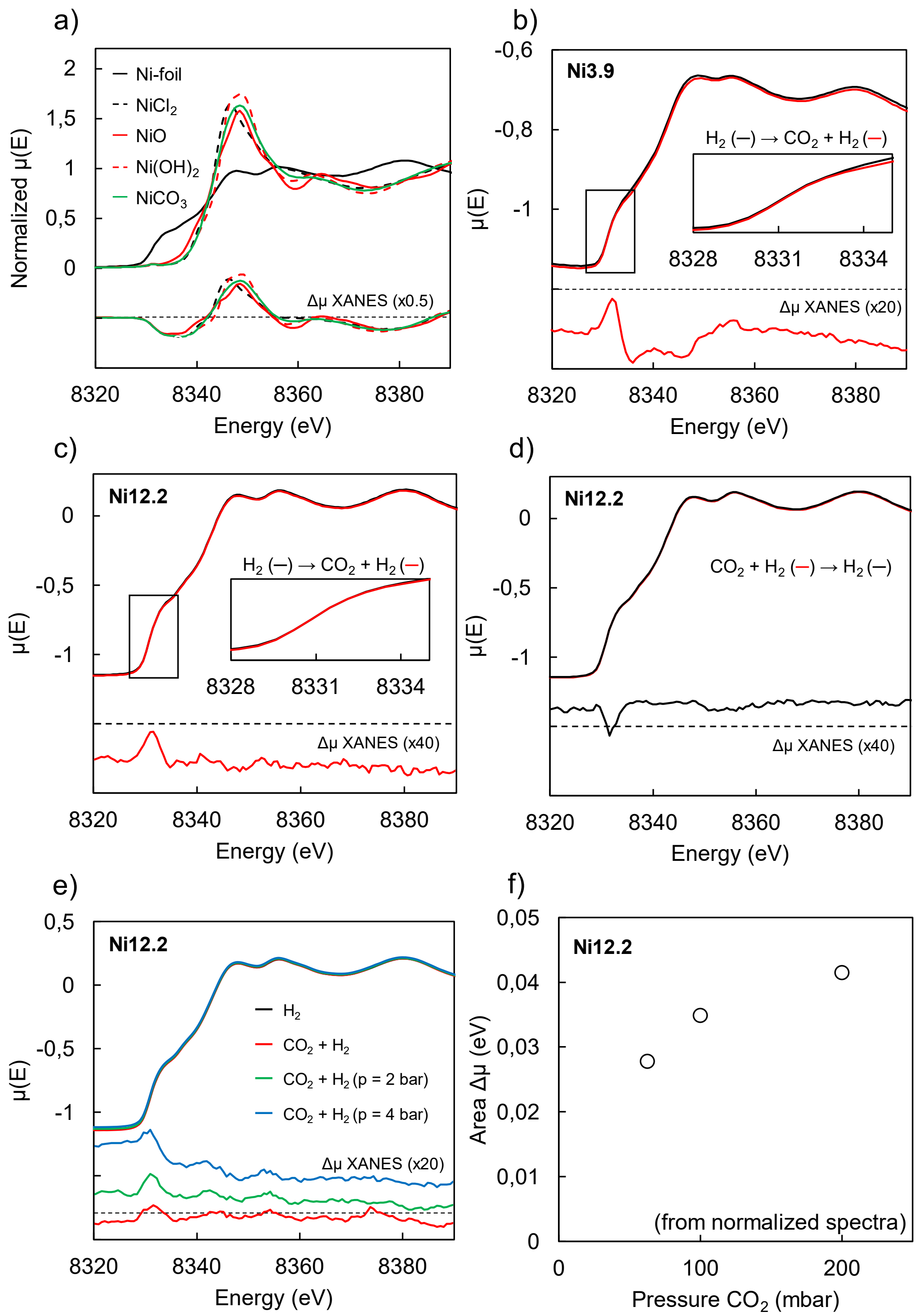

Figure S12. a) XANES results of Ni reference materials. The difference with Ni-foil is shown below the XANES spectra. b) Raw XANES data of the Ni3.9 sample obtained before and after the switch from H_2 to CO_2 + H_2 (50 ml/min, 5 vol% CO_2 and 20 vol% H_2 in Ar, 220 °C). As shown in the Δμ results below, the introduction of CO_2 results in an increase in intensity of the low-energy part of the edge and in a shift of the edge position to higher energies. c) Raw XANES data of the Ni12.2 sample obtained before and after the switch from H_2 to CO_2 + H_2 d) Raw XANES data of the Ni12.2 sample of the switch from CO_2 + H_2 back to H_2. e) Raw XANES data of the Ni12.2 sample for increasing CO_2 hydrogenation pressures (1, 2 and 4 bar). f) Integrated changes in Δμ area from normalized XANES spectra as a function of CO_2 pressure obtained from increasing CO_2 hydrogenation pressures from 1 to 4 bar.

Infrared spectroscopy

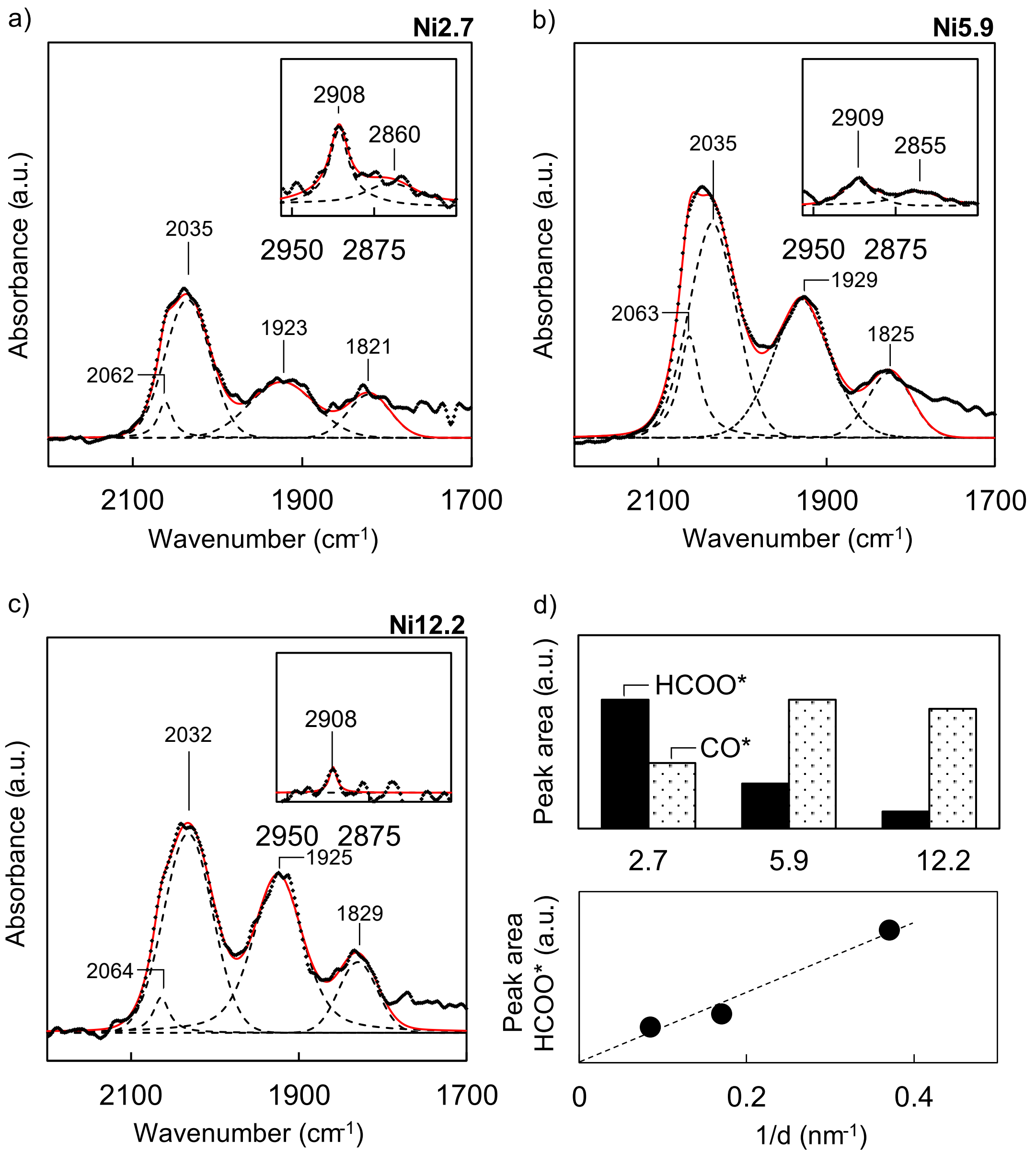

Figure S13. DRIFTS spectra obtained during CO_2 hydrogenation at 200°C of the catalyst sample a) Ni2.7, b) Ni5.9 and c) Ni11.9. For all figures, the same y-axis has been used. The inserts contain the spectra of the C-H region and the same y-axis has been used for the inserts as well. The spectra are corrected for the differences in optical pathlength by using the overtone and combination vibrations of silica in the 2100-1800 cm^{-1} region.[109,110] In addition, the spectra are normalized by Ni surface area as determined from H_2-chemisorption. d) Relative peak area of the HCOO* (2910 cm^{-1}) and CO* (2060+2035+1925+1825 cm^{-1}) species (top figure) and Peak area of band corresponding to HCOO* (2910 cm^{-1}) as a function of $1/d_{NP}$.

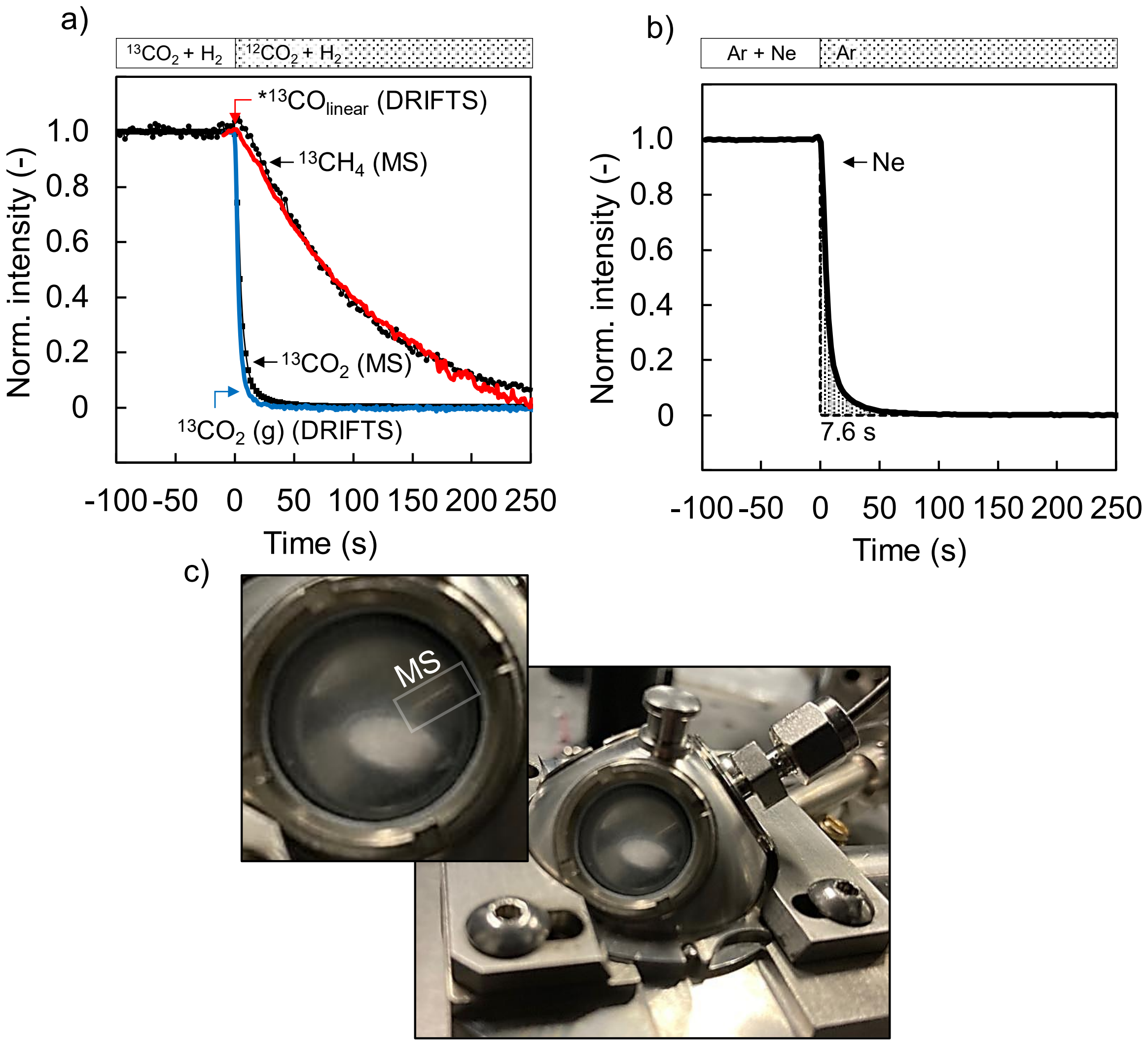

Figure S14. Optimized DRIFTS-MS setup for transient experiments. a) DRIFTS-MS results during $^{13}CO_2 + H_2$ to $^{12}CO_2 + H_2$ switch. The $^{13}CH_4$ and $^{13}CO_2$ transients from MS correspond well with the linear carbonyls and gas-phase CO_2 transients obtained from DRIFTS. b) Tracer experiment in the DRIFTS-MS setup using Ne as a tracer. c) Photograph of the modified DRIFTS cell. The inlet of the MS capillary is positioned close to the catalyst bed. The reaction mixture was introduced at the bottom of the sample cup. This configuration allows fast gas replacement during switches, with an equal gas hold-up times obtained from both IR and MS.

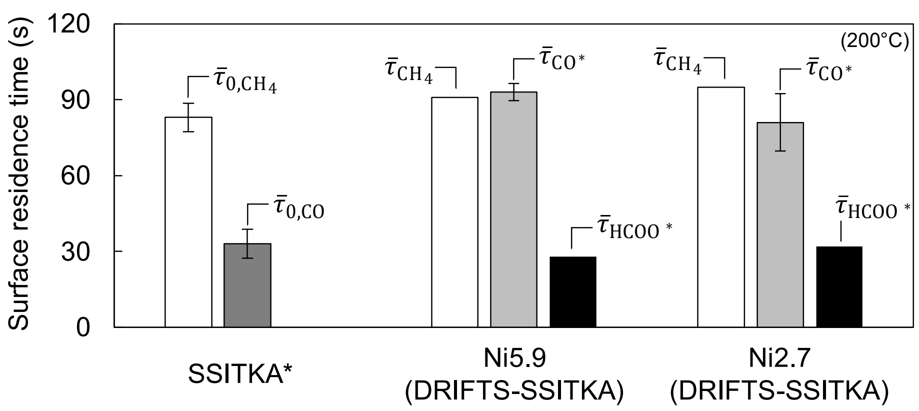

Figure S15. Surface residence times from SSITKA during CO_2 hydrogenation in fixed bed reactor, denoted as 'SSITKA', and from DRIFTS-SSITKA experiments for Ni5.9 and Ni2.7 samples. (*) The surface residence times for the fixed bed reactor have been calculated with the τ_0 values obtained at 220 °C, using the apparent activation energies for CH_4 (91 kJ/mol) and CO (68 kJ/mol) formation.

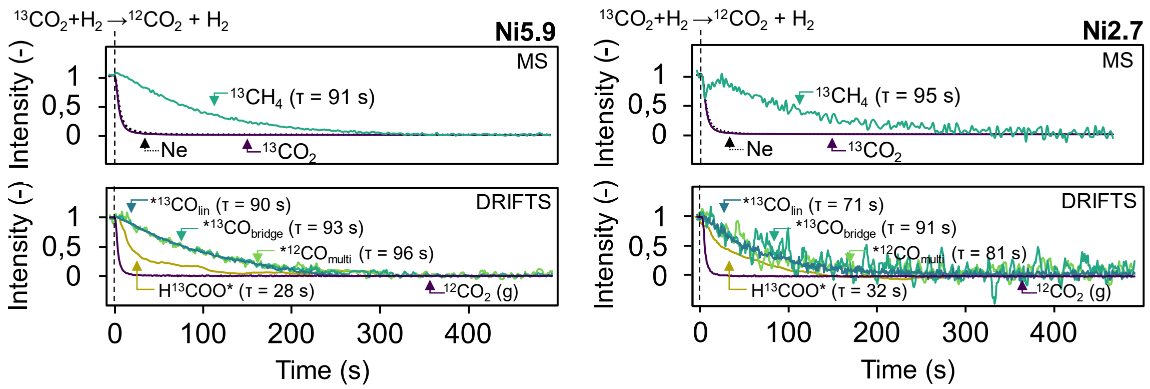

Figure S16. DRIFTS-SSITKA results of Ni5.9 (left panels) and Ni2.7 (right panels) for $^{13}CO_2$ to $^{12}CO_2$ switch during CO_2 hydrogenation conditions (50 mL/min 5 vol% CO_2 and 20 vol% H_2 in Ar at 220 °C). Only the ^{13}C-labelled species are shown.

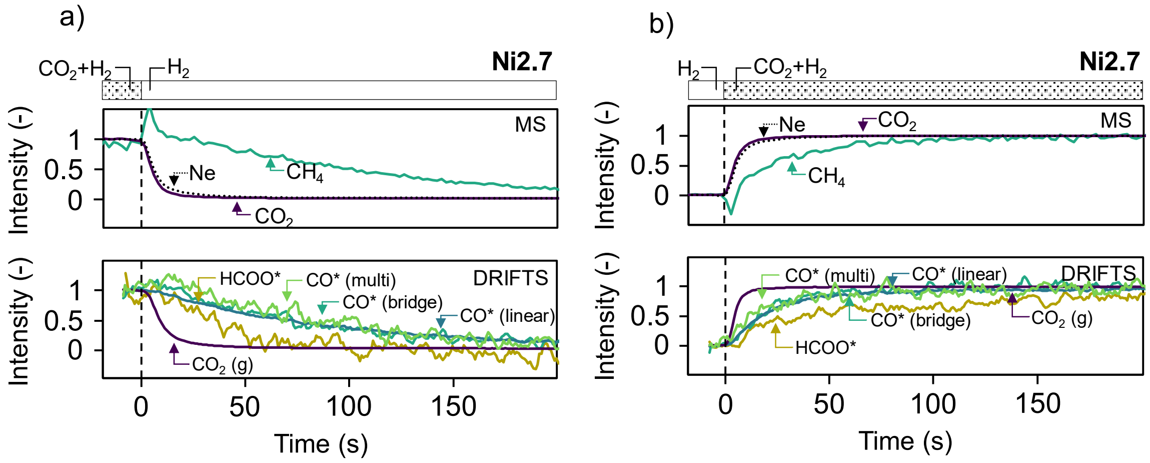

Figure S17. DRIFTS-MS results of Ni2.7 during switches from CO_2 + H_2 to H_2 (a) and H_2 to CO_2 + H_2 (b) during CO_2 hydrogenation conditions at 200 °C.

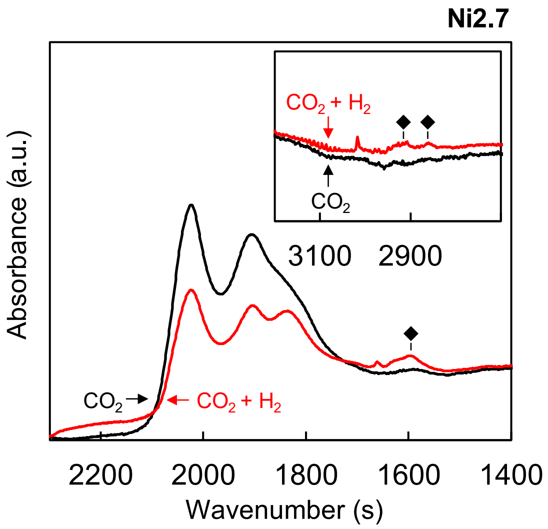

Figure S18. Transmission FTIR spectra for Ni2.7 at 220°C and 10 mbar CO_2 (black line) and 10 mbar CO_2 + 40 mbar H_2 (red line). The catalyst has been pretreated in H_2 at 550°C, evacuated and cooled down to 220°C in vacuum before exposing the surface to CO_2. The insert contains the spectra between 2800-3150 cm^{-1}. Diamond symbols indicate the absorption bands of formate species.

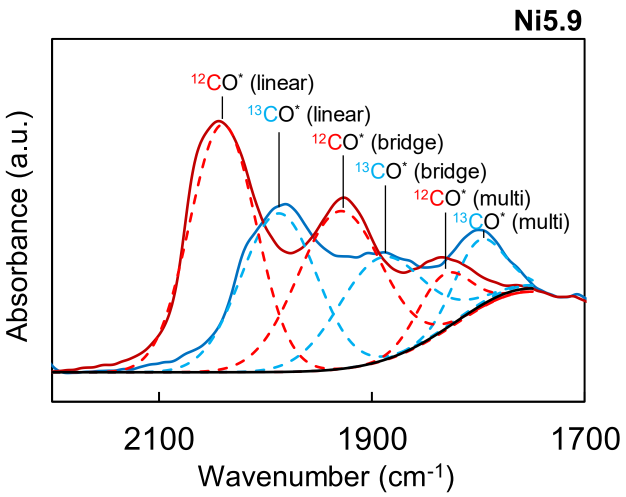

Figure S19. Example of DRIFTS-SSITKA result during steady-state $^{12}CO_2$ (solid red line) and $^{13}CO_2$ (solid blue line) hydrogenation. The dotted lines are the fitted lines for linear, bridged and multibonded carbonyl species. To obtain the residence times of the carbonyl species during SSITKA switches, the spectra were fitted by using the steady-state fits as shown below. For example $y_{linear}(t) = c(t) * y_{fit,12C\ linear} + (1-c(t)) * y_{fit,\ 13C\ linear}$, where c is relative concentration of linear ^{12}C-carbonyl species.

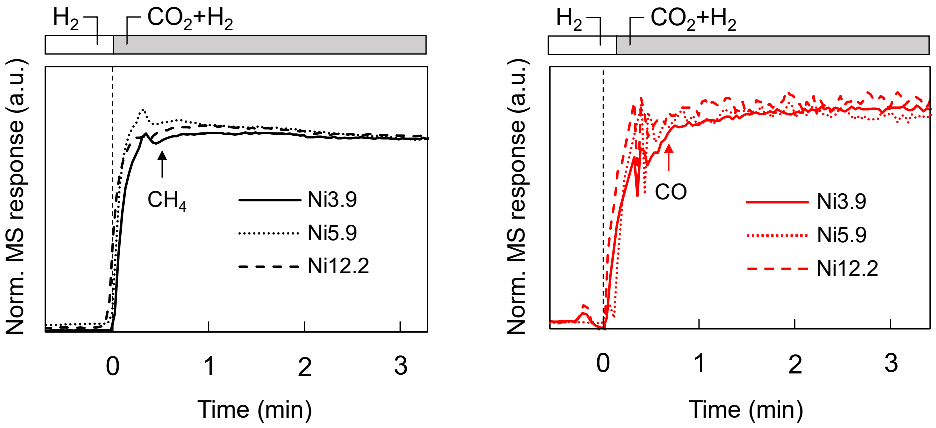

Figure S20| Normalized MS response of CH_4 (m/z = 15) and CO (m/z = 28) during switch from H_2 to CO_2 + H_2 for Ni3.9, Ni5.9 and Ni12.2.

Note S4. Thermodynamic equilibrium of RWGS and methanation.

Thermodynamic calculations (Figure S21) show that under applied reaction conditions (T = 220 °C) the equilibrium conversion of CO_2 is 14 % for RWGS and 97 % for methanation, which is well above the conversion levels used in this work. The approach to equilibrium (η),

$$\eta_{RWGS} = \frac{P_{CO}P_{H_2O}}{P_{CO_2}P_{H_2}}\frac{1}{K_{eq,RWGS}} \tag{1}$$

$$\eta_{methanation} = \frac{P_{CH_4}P_{H_2O}^2}{P_{CO_2}P_{H_2}^4}\frac{1}{K_{eq,methanation}} \tag{2}$$

$$r_{net} = r_{forward}(1-\eta) \quad ` \tag{3}$$

where $K_{eq,RWGS}$ and $K_{eq,methanation}$ are the equilibrium constants for the RWGS and methanation reactions, can be used to correct the observed rates for the approach to equilibrium. For the reaction conditions used in this study (220°C, 5 kPa CO_2 and 20 kPa H_2, 1.5-1.8 % conversion, 40-90 % selectivity to CH_4), η_{RWGS} is 0.002-0.008 and $\eta_{methanation}$ is $<10^{-6}$, which confirms that the conversion of CO_2 is far from equilibrium and that no correction is required.

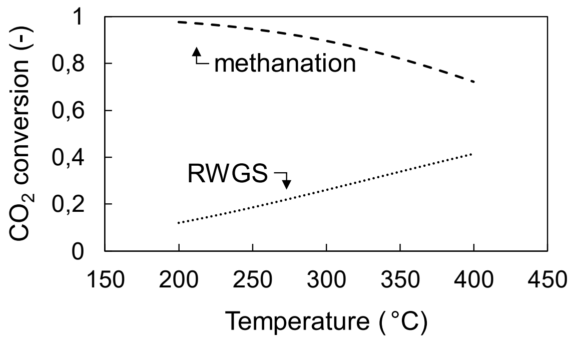

Figure S21| Equilibrium conversion for RWGS and methanation reactions (5 kPa CO_2 + 20 kPa H_2, 1 bar, T = 200–400 °C).

Note S5. Mass- and heat-transfer limitations.

We checked the absence of mass- and heat-transfer limitations in the reactor with the following calculations and experiments:

Axial dispersion

The criterion for allowing the assumption of plug-flow behaviour is given by[12,13]:

$$\frac{h_{bed}}{d_p} > \frac{8}{Bo} n \ln\left(\frac{1}{1-X_{CO_2}}\right) \tag{1.1}$$

in which:	h_{bed}	bed length	80	(mm)
	d_p	particle diameter	0.18	(mm)
	Bo	Bodenstein number	0.63	(-)
	n	reaction order	1	(-)
	X_{CO_2}	conversion of CO_2	0.03	(-)

The Bodenstein number is obtained from the correlation[14]:

$$\frac{1}{Bo} = \frac{\epsilon_b}{\tau_b Re Sc} + 0.5 \tag{1.2}$$

in which:	ϵ_b	bed porosity	0.40	(-)
	τ_b	bed tortuosity	1.58	(-)
	Re	Reynolds number	0.43	(-)
	Sc	Schmidt number	0.55	(-)

Tortuosity τ_b [15], Reynolds number Re and Schmidt number Sc are given by:

$$\tau_b = \frac{1}{\sqrt{\epsilon_b}} \tag{1.3}$$

$$Re = \frac{d_p \rho_G u_0}{\mu_G} \tag{1.4}$$

$$Sc = \frac{\mu_G}{\rho_G D_{CO_2,m}} \tag{1.5}$$

in which:	ρ_G	gas mixture density	0.80	($kg \cdot m^{-3}$)
	u_0	superficial velocity	0.078	(m/s)
	μ_G	gas mixture viscosity	$2.6 \cdot 10^{-5}$	($kg \cdot m^{-1} \cdot s^{-1}$)
	$D_{CO_2,m}$	diffusion coefficient of CO_2 in gas mixture	$6.0 \cdot 10^{-5}$	(m^2/s)

Substituting the parameters in the equations above leads to the criterion $\frac{h_{bed}}{d_p} > 0.34$. Since the ratio between h_{bed} and d_p is 444, the assumption of plug-flow behaviour can be justified.

Radial dispersion

The assumption of the absence of radial concentration gradients is valid according to the criterion:

$$\frac{d_t}{d_p} > 8 \tag{2.1}$$

in which:	d_t	catalyst bed diameter	5	(mm)
	d_p	particle diameter	0.18	(mm)

Since d_t/d_p is 28, the radial concentration gradient can be assumed to be negligible.

External mass transfer

External mass transfer limitations are considered absent if the Mears' criterion is obeyed[16], expressed using the Carberry number Ca:

$$Ca = \frac{R^{obs}_{v,CO_2}}{k_g a_v C_{CO_2,b}} < \frac{0.05}{n} \tag{3.1}$$

in which:	R^{obs}_{v,CO_2}	Volumetric reaction rate per unit of catalyst pellet volume	0.3	($mol \cdot m^{-3} \cdot s^{-1}$)
	k_G	External mass transfer coefficient	0.84	(-)
	a_v	External surface area ($6/d_p$)	$3.3 \cdot 10^4$	($m^2 \cdot m^{-3}$)
	$C_{CO_2,b}$	Bulk CO_2 concentration	1.22	($mol \cdot m^{-3}$)

External mass transfer coefficient k_G is obtained by using the correlation[14]:

$$Sh = \frac{k_G d_p}{D_{CO_2,m}} = 2 + 1.1 Re^{0.6} Sc^{1/3} \tag{3.2}$$

in which:	Sh	Sherwood number	2.54	(-)

From the parameters above, a Ca number of $8.7 \cdot 10^{-6}$ is obtained and with this, the Mears' criterion is obeyed.

Internal mass transfer

The extent of internal mass transfer is expressed using the Weisz modulus Φ and is defined as[17]:

$$\Phi = \left(\frac{n+1}{2}\right) \frac{R^{obs}_{v,CO_2} \left(\frac{1}{a_v}\right)^2}{D_{CO_2,eff} C_{CO_2,s}} < 0.08 \tag{4.1}$$

in which:	$D_{CO_2,eff}$	effective diffusivity in catalyst particle	$3.92 \cdot 10^{-7}$	($m^2 \cdot s$)
	$C_{CO_2,s}$	concentration of CO_2 at catalyst surface	1.22	($mol \cdot m^{-3}$)

In absence of external diffusion limitations, the CO_2 concentration near the catalyst surface equals the bulk concentration. This results in a Weisz modulus of $5.6 \cdot 10^{-4}$, which is much smaller the criterion of 0.08.

Radial heat transfer

According to Mears[16], the radial heat transfer gradient ΔT_{rad} is negligible when:

$$\Delta T_{rad} = \frac{R^{obs}_{v,CO_2} |\Delta_r H| (1-\epsilon_b) \rho_b (1-b) d_t^2}{32 \lambda_{er}} < \frac{0.05 R T_w^2}{E_a} \tag{5.1}$$

in which:	$\Delta_r H$	reaction enthalpy	-165	($kJ \cdot mol^{-1}$)
	b	volume fraction of inert material	0.82	(-)
	λ_{er}	effective radial thermal conductivity in the bed	1.53	($W \cdot m^{-1} \cdot K^{-1}$)
	R	gas constant	8.31	($J \cdot mol^{-1} \cdot K^{-1}$)
	T_w	reactor wall temperature	493	(K)

$E_{a,app}$	apparent activation energy	90	(kJ·mol^{-1})

The average pellet heat conductivity λ_p is calculated with:

$$\frac{1}{\lambda_p} = \frac{1-b}{\lambda_{cat}} + \frac{b}{\lambda_{SiC}} \tag{5.2}$$

where b is the volumetric fraction of SiC (0.82), λ_{cat} is the conductivity of the catalyst particles (~0.5 W m^{-1} K^{-1}) and λ_{SiC} is the conductivity of SiC (~40 W m^{-1} K^{-1}). According to Specchia[18], the effective radial thermal conductivity in the bed λ_{er} can be estimated by:

$$\frac{\lambda_{er}}{\lambda_g} = \frac{\lambda_{stat}}{\lambda_g} + \frac{\lambda_{conv}}{\lambda_g} \tag{5.3}$$

$$\frac{\lambda_{stat}}{\lambda_g} = \varepsilon_b + \frac{1-\varepsilon_b}{0.220\varepsilon_b^2 + \frac{2}{3}\left(\frac{\lambda_g}{\lambda_p}\right)} \tag{5.4}$$

$$\frac{\lambda_{conv}}{\lambda_g} = \frac{Re\ Pr}{8.65\left(1+19.4\left(\frac{d_p}{d_t}\right)^2\right)} \tag{5.5}$$

where λ_g is the conductivity of the gas mixture and λ_{stat} and λ_{conv} are the static and convective contributions to radial thermal conductivity.

A value for ΔT_{rad} of 2.7·10^{-3} K is obtained, which is lower than criterion of 1.12 K.

Adiabatic temperature rise

The adiabatic temperature rise ΔT_{ad} is calculated from:

$$\Delta T_{ad} = \frac{|\Delta_r H| y_{CO_2} X_{CO_2}}{C_{p,G}} \tag{6.1}$$

in which:	y_{CO_2}	molar fraction CO_2	0.05	(-)
	$C_{p,G}$	heat capacity gas mixture	110	(J·mol^{-1}·K^{-1})

Since ΔT_{ad} is 2.0 K, which is small compared to the used reaction temperature, the temperature rise along the reactor bed is negligible.

Experimental tests

Varying the reactant mixture flow between 20-70 ml min^{-1} while keeping the CO_2/H_2 ratio constant resulted in similar CH_4 formation rates (Figure S22b), which indicates that the observed rates are not affected by external diffusion limitations under typical reaction conditions (50 mL/min, 5 kPa CO_2 and 20 kPa H_2 in Ar). To assess the presence of internal mass transfer limitation, we measured the CO_2 hydrogenation rates of a Ni/SiO_2 catalyst with different catalyst particle size (Figure S22c). For sizes below 250 μm, the observed rate for the formation of CH_4 remains constant. Since a catalyst fraction between 125-250 μm is used for all catalytic tests, we confirm the absence of internal mass transfer limitations for the conditions used in this study.

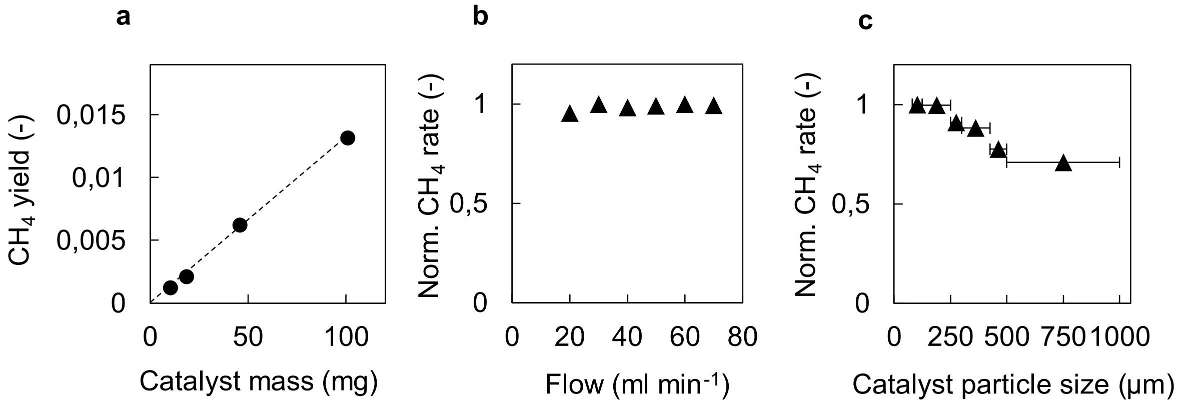

Figure S22| **a**, CH_4 yield versus catalyst mass (Ni12.2) during CO_2 hydrogenation (5 kPa CO_2 and 20 kPa H_2 in Ar, 220 °C, 125-250 μm). **b**, Normalized rate of CH_4 formation as a function of reactant mixture flow (5 kPa CO_2 and 20 kPa H_2 in Ar, 220 °C, 0.9-1.8 % conversion, 125-250 μm). **c**, Normalized CH_4 formation rate versus catalyst particle size (50 ml/5 kPa CO_2 and 20 kPa H_2 in Ar, 220 °C, 1.9-2.8 % conversion). The rates are normalized by the maximum rate obtained during the test.

Note S6. Selectivity-conversion correlation for CO_2 hydrogenation.

The correlation between conversion and selectivity as observed for CO_2 hydrogenation is due to the sequential nature of this reaction. As the formation of CO precedes the hydrogenation of CO to CH_4, CO formed at the beginning of the catalyst bed is likely converted to CH_4 further down the bed. The CO formed at the end of the bed, however, will have a lower probability of being hydrogenated to CH_4.

Assuming the methanation of CO_2 follows a A→B→C reaction path, the rates can be defined as

$$r_A = -k_1 p_A \quad (1)$$

$$r_B = +k_1 p_A - k_2 p_B \quad (2)$$

$$r_C = +k_2 p_B \quad (3)$$

where k_1 and k_2 are the rate constants for reactions A→B and B→C, and p_x is the partial pressure of component x. By solving these equations for a plug flow reactor (PFR) with appropriate boundary conditions, the partial pressures of A, B and C are given by:

$$p_A(\tau) = p_{A,0} e^{-k_1 \tau} \quad (4)$$

$$p_B(\tau) = p_{B,0} e^{-k_2 \tau} + \frac{k_1 p_{A,0}}{k_2 - k_1}\left(e^{-k_1 \tau} - e^{-k_2 \tau}\right) \quad (5)$$

$$p_C(\tau) = p_{C,0} + p_{B,0}\left(1 - e^{-k_2 \tau}\right) + \frac{p_{A,0}}{k_2 - k_1}\left(k_2\left(1 - e^{-k_1 \tau}\right) - k_1\left(1 - e^{-k_2 \tau}\right)\right) \quad (6)$$

An example of the concentration profiles of A, B and C as a function of residence time τ in a PFR are shown in Figure S23 below. In our case, the conversion is low (0.015-0.018) and therefore, a lower CH_4 selectivity is expected. An example of the relation between conversion and selectivity of our largest and smallest Ni nanoparticles in included in the figure. Note that when measuring the rates at increasing CO_2 conversions, the differences between CO and CH_4 selectivity for the different samples will gradually disappear. The same holds for measuring the rates at high temperatures: with increasing temperature, the k_2/k_1 ratio increases ($E_{a,app,RWGS}$ ~ 70 kJ/mol, $E_{a,app,COmethanation}$ ~ 90 kJ/mol), which results in a higher CH_4 selectivity. This example demonstrates the importance of evaluating the CO_2 hydrogenation performance of catalysts at similar (and low) conversion values to properly probe the differences in RWGS and CO methanation activity.

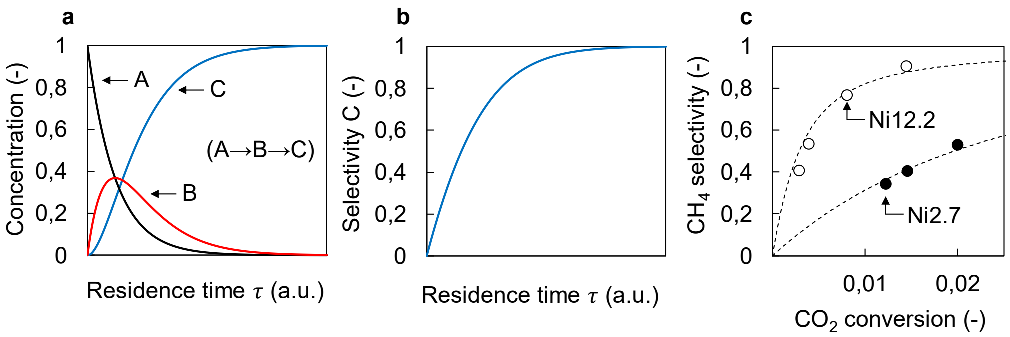

Figure S23| a, Concentration of A, B and C versus residence time, using the model described in the text (with k_1 being equal to k_2). **b**, Selectivity of C versus residence time. **c**, CH_4 selectivity versus CO_2 conversion for the Ni2.7 (solid circles) and Ni12.2 (open circles) samples during CO_2 hydrogenation (220 °C, 50 ml/min, 5 kPa CO_2 and 20 kPa H_2 in Ar). The amount of catalyst in the reactor is varied to obtain different conversions. The dashed lines present the model described in the text above, using different k_2/k_1 ratios.

Note S7. Particle size effects for CO_2 and CO hydrogenation.

In the tables shown below, we have included an overview of previous studies on the particle size effects for CO_2 (Table S4) and CO (Table S5) hydrogenation. From the trends presented in Table S4, it becomes clear that the effect of nanoparticle size on the catalytic activity of CO_2 hydrogenation to CO and CH_4 is still subject of debate. A number of research groups found increasing surface-specific CO_2 conversion rates when increasing particle size[4,6,8,14,16,17,24,25] or decreasing particle size[1,5,7,9,12], while others observed an optimum as a function of particle size[2,26,27], and few reported that there was no particle size effect at all[15,18]. Some studies report high CH_4 selectivity for small metal nanoparticles[2,13,15,18,25-27], whereas many others note that CO is the dominant product for small metal entities and CH_4 selectivity increases when increasing the particle size.[1,5,7,8,11,13,30,31] Wu et al.[1] observed a sharp decrease in CO_2 conversion rates when increasing the Ni weight loading from 0.5 to 10 %, while the rates corresponding to CH_4 formation increased. The authors attribute the differences in catalytic activity to the presence consecutive and parallel reaction pathways for the production of CO and CH_4. Small particles display high CO_2 conversion rate and a low selectivity to CH_4, which is linked to the decomposition of formate to CO and the consecutive hydrogenation of CO to CH_4. The increased CH_4 selectivity for large particles is linked to the direction methanation of formate species to CH_4 instead of CO through a parallel reaction pathway. Vogt et al.[2] observed a volcano-type trend for the surface-specific rate of CH_4 formation as a function of Ni nanoparticle size, with particles of 3-4 nm giving the highest activity. The binding strength of CO is argued to be a key descriptor for the catalytic performance. Larger particles tend to bind CO too strong, resulting in the inhibition of the reaction by adsorbed CO. Conversely, smaller particle bind CO too weak, which is attributed to the increased susceptibility of these nanoparticle surfaces to oxidation during the hydrogenation of CO_2. In contrast to the studies of Wu and Vogt, Beierlein et al.[3] found the methanation rates to be invariant of the Ni nanoparticle size, and argued that the methanation of CO_2 is an structure insensitive reaction. These contradictions illustrate the persistent ambiguity about structure-performance relationships for CO_2 hydrogenation reactions, which raise various questions. First, discussions about the mechanism of CO_2 hydrogenation generally revolve around two mechanistic proposals: 1) a consecutive reaction pathway with CO as a key intermediate, or 2) a reaction pathway where CO_2 is directly converted into CH_4 without the intervening formation of CO. While metal-support interfaces are sometimes thought to give access to different mechanisms[34], the first reaction mechanism is often considered as the dominant one for Ni nanoparticles supported by SiO_2 or Al_2O_3[2,5,8,15-17,24,30,31]. In case of this consecutive mechanism, one would expect similarities between particle size effects found for CO methanation (Table S5), but clear examples of similar size effects for CO_2 and CO methanation are difficult to find in literature. As a possible explanation for the difference with CO methanation, some have suggested that CO_2 can induce changes of the nanoparticle surface.[2,8,26,32] These changes can include oxidation, restructuring and poisoning, which have been used to interpret the observed particle size effects. In our work, we have studied both mechanistic details of

CO_2 hydrogenation and the structural characteristics of the nanoparticles under reaction conditions, in order to gain insights into the much debated particle size effect of CO_2 hydrogenation.

Although the purpose of our work is not to discredit the works of others that report different particle size dependencies, we can provide some explanations for the observed discrepancies. As shown in Table S4, some studies use a small number of catalysts, sometimes even only 2 samples, and a relatively narrow range of particle sizes to study the size effect of CO_2 hydrogenation. Generally, strong variations in surface-specific are often found when the particles are in the 1-7 nm range[1-11,13,14,16,17], while for studies that include larger nanoparticles such variations are less pronounced[12,15,18]. Therefore, we argue that it is necessary to use a large enough set of catalysts that contains particles at least the 1-15 nm range to map particle size effects. Another issue can be found in the use of relatively high temperatures up to 400 °C and CO_2 conversion levels above 10% to determine reaction rates for CO_2 hydrogenation. Under these conditions, it is essential to confirm the absence of limitations due to mass/heat transfer and to properly consider the approach to thermodynamic equilibrium of the RWGS and methanation reactions. Particularly, when the difference in conversion rates between samples is significant , these limitations could considerably affect the particle size versus activity trends. As discussed in note S6, the conversion should be kept constant to avoid deviations in selectivity due to conversion-selectivity correlations. For the determination of the metal surface area, which is required for obtaining surface-specific rates, chemisorption and TEM are the most popular methods. While both methods can give reasonably accurate dispersion values, a sufficient amount of particles per sample (500 or more[33]) needs to be analyzed. Moreover, the degree of reduction after H_2-pretreatment should be known when TEM is used to estimate the metal surface area. Most preferably, multiple methods are to be used to determine the metal surface area and the particle size. Finally, kinetic parameters besides surface-specific rates, for example apparent activation energy, reaction orders and mean surface residence time, can provide important insights into the relation between activity and particle size.

References used in Note S7, and Tables S4 and S5:

[1] Wu H.C. et al. (2015) *Catal. Sci Technol.* **5**, 4154-4163

[2] Vogt C. et al. (2018) *Nat. Catal.* **1**, 127-134

[3] Pu T. et al. (2021) *J. Catal.* **400**, 228-233

[4] Zheng H. et al. (2022) *ACS Catal.* **12**, 15451-15462

[5] Chen C.S. et al. (2017) *ACS Catal.* **7**, 8367-8381

[6] Lin L. et al. (2021) *J. Energy Chem.* **61**, 602-611

[7] Budi C.S. et al. (2016) *ChemSusChem* **9**, 2326-2331

[8] Feng K. et al. (2021) *Appl Catal. B* **292**, 120191

[9] Wang K. et al. (2021) *Fuel* **304**, 121388

[10] Pu T. et al. (2021) *AIChE* **68**, e17458

[11] Winter L.R. et al. (2018) *Appl. Catal. B* **224**, 442-450

[12] Hao Z. et al. (2021) *Appl. Catal. B* **286**, 119922

[13] Botzolaki G. et al. (2020) *Catalysts* **10**, 944

[14] Zhu J. et al. (2020) *ACS Catal.* **10**, 7424-7433

[15] Karelovic A. et al. (2012) *Appl Catal B* **113-114**, 237-249

[16] Iablokov V. (2012) *Nano Lett.* **12**, 3091-3096

[17] Visser N. et al. (2022) *ChemCatChem* **14**, e202200665

[18] Beierlein et al. (2019) *Appl. Catal. B* **247**, 200-219

[19] Bezemer G.L. et al. (2006) *J. Am. Chem. Soc.* **128**(10), 3956

[20] Xiong H. (2011) *J. Catal.* **278**, 26-40

[21] Yang J. et al. (2010) *Langmuir* **26**, 16558-16567

[22] Park J.Y. et al. (2010) *J. Molecular Catal. A **323***, 84-90

[23] Bartholomew C.H. et al. (1981) *Ind. Eng. Chem. Prod. Res. Dev.* **20**, 296-300

[24] Kwak J.H. et al. (2013) *ACS Catal.* **3**, 2449-2455

[25] Panagiotopoulou P. et al. (2017) *Appl. Catal. A Gen.* **542**, 63-70

[26] Vogt C. et al. (2021) *Nat. Comm.* **12**, 7096

[27] Guo Y. et al. (2018) *ACS Catal.* **8**, 6203-6215

[30] Aitbekova A. et al. (2018) *J. Am. Soc. Chem.* **140**, 13736-13745

[31] Matsubu J.C. et al. (2015) *J. Am. Chem. Soc.* **137**, 3076-3084

[32] De Coster V. et al. (2022) *J. Phys. Chem. Lett.* **13**, 7947-7952 (2022)

[33] Rice S.B. et al. (2013) *Metrologia* **50**, 663-678

[34] Kattel S. et al. (2017) *J. Am. Chem. Soc.* **139**, 9739-9754

Table S4| Particle size effects for CO_2 hydrogenation

Authors	Catalyst	Particle sizes (nm)	Loading (%)	Conditions	Dispersion	r_{surf} vs d
Wu H.C. et al.[1]	Ni/SiO_2	?, 9	0.5, 10	350 °C<br>1:1 CO_2 :H_2<br>conversion ?	CO-chem.	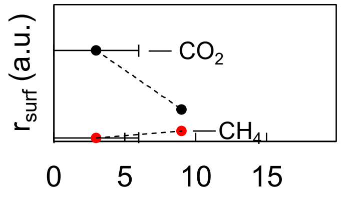
Vogt C. et al. [2]	Ni/SiO_2	1.2-6.9	1-60	400 °C<br>1:4 CO_2:H_2<br>conversion ?	TEM	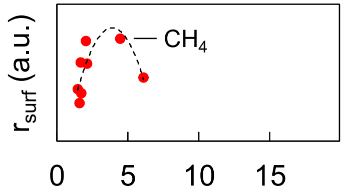
Pu T. et al[3]	Ni/SiO_2	2.7, 11.5	10, 40	400 C<br>1:4 CO_2:H_2<br>conversion ?	-	n.a.
Zheng H. et al.[4]	Ni/CeO_2	?, 2.1, 4.2	0.1-5	290 °C<br>1:4 CO_2:H_2<br>conversion < 10 %	TEM	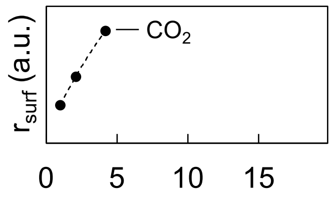
Chen C.S. et al.[5]	Ni/SBA	2.7, 3.6, 4.7	5.9-21.7	350 °C<br>1:1 CO_2:H_2<br>conversion ?	H_2-chem.	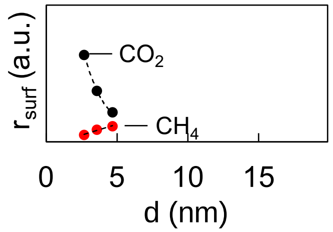

(continued)

**Authors**	**Catalyst**	**Particle sizes (nm)**	**Loading (%)**	**Conditions**	**Dispersion**	**r_{surf} vs d**
Lin L. et al.[6]	Ni/CeO_2	2.1, 4.2, 7.8	2	250 °C<br>1:4 CO_2:H_2<br>conversion 0.4-3.2 %	TEM	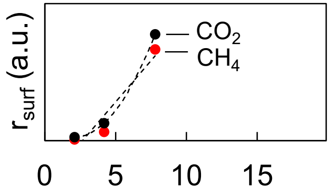
Budi C.S. et al.[7]	Ni/SBA	6.5, 7.4, 8.9	3.3-12.0	400 °C<br>1:1 CO_2:H_2<br>conversion <10 %	TEM	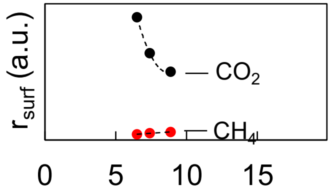
Feng K.[8]	Ni$_x$/Mg$_{0.9}$Ni$_{0.1-x}$Al$_2$O$_4$	0.9-12.3	3.3 (mol%)	400 °C<br>1:4 CO_2:H_2<br>conversion 5-80 %	TEM	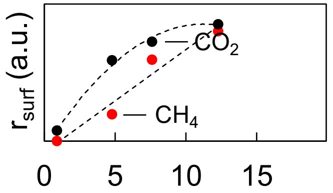
Wang K. et al.[9]	Ni/SiO_2	3.5-7.5	2	250 °C<br>1:4 CO_2:H_2<br>conversion ?	H_2-chem.	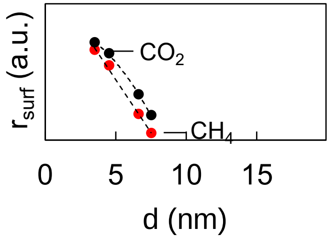
Pu T. et al.[10]	Ni/SiO_2	1.8-5.2	10-40	350 °C<br>1:4 CO_2:H_2<br>conversion 1.5-3.5 %	-	n.a.

(continued)

**Authors**	**Catalyst**	**Particle sizes (nm)**	**Loading (%)**	**Conditions**	**Dispersion**	**r_{surf} vs d**
Winter et al.[11]	Ni/CeO_2	0.64-1.12	1-10	350 °C<br>1:3 CO_2:H_2<br>conversion 10 %	CO-chem.	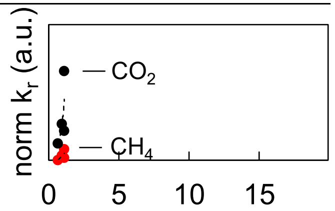
Hao et al.[12]	Ni/CeO_2	8.3-21.0 nm	1-10	275 °C<br>1:4 CO_2:H_2<br>conversion ?	TEM	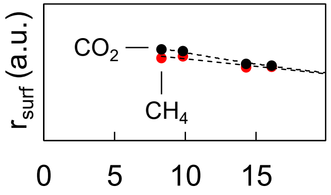
Botzolaki et al.[13]	Rh/Al_2O_3<br><br>$Rh/CeZrO_x$	1.2, 1.6, 2.1<br><br>2.1, 2.3, 5	2	280 °C<br>1:4 CO_2:H_2<br>conversion 5-15 %	H_2-chem./TEM	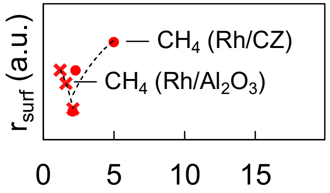
Zhu J. et al.[14]	FeC/ZrO_2	2.5-12.9 nm	10	320 °C<br>1:4 CO_2:H_2<br>30 bar<br>conversion ~12 %	CO-chem.	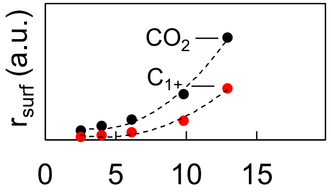
Karelovic A. et al.[15]	Rh/Al_2O_3	3.6-15.4 nm	1-5	200 °C<br>1:4 CO_2:H_2<br>conversion < 10 %	H_2-chem.	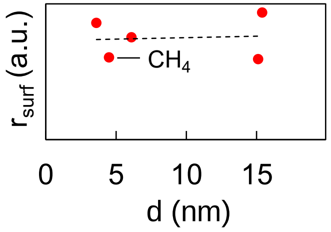

(continued)

**Authors**	**Catalyst**	**Particle sizes (nm)**	**Loading (%)**	**Conditions**	**Dispersion**	**r_{surf} vs d**
Iablokov V. et al.[16]	Co/SiO_2	3, 7, 10	1.20-2.77	250 °C<br>1:3 CO_2:H_2<br>6 bar<br>conversion <10 %	TEM	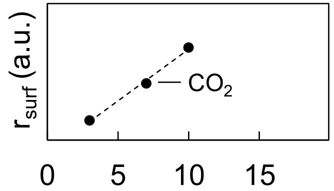
Visser N. et al.[17]	Ni/OxC	4-8	4.3-11.6	300 °C<br>1:4 CO_2:H_2<br>conversion ~20%	TEM	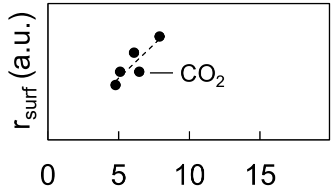
Beierlein et al.[18]	Ni/Al_2O_3	5-91	14-88	300 °C<br>1:4 CO_2:H_2<br>conversion ~30 %	H_2-chem.	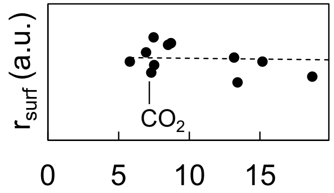
Simons et al. (this work)	Ni/SiO_2	2.7-12.2	1.7-14.6	220 °C<br>1:4 CO_2:H_2<br>conversion 1.5-1.8 %	H_2-chem.	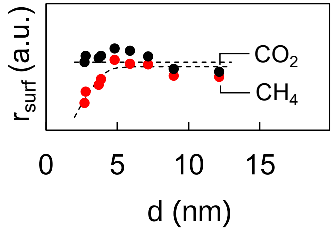

Table S5| Particle size effects for CO hydrogenation

Authors	Catalyst	Particle sizes (nm)	Loading (%)	Conditions	Dispersion	r_{surf} vs d
Bezemer et al.[19]	Co/C	3-27	0.8-22	220 °C<br>1:2 CO :H_2<br>1 bar<br>conversion 2 %	XPS/H_2-chem.	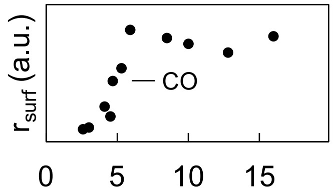
Xiong H. et al. [20]	Co/C	3-45	1-13	225 °C<br>1:2 CO:H_2<br>8 bar<br>conversion 3-26 %	H_2-chem.	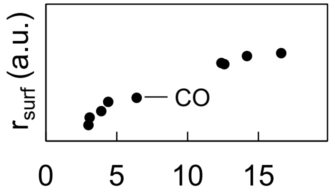
Yang et al.[21]	Co/Al_2O_3	4-11	20	210 C<br>1:10 CO:H_2<br>1.85 bar<br>conversion ?	H_2-chem.	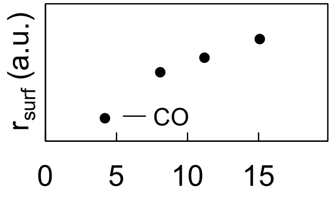
Park et al.[22]	Fe/Al_2O_3	2-8	5	280 °C<br>1:2 CO_2:H_2<br>10 bar<br>conversion 6-32 %	CO-chem.	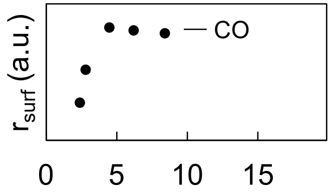
Bartholomew C.H. et al.[23]	Ni/Al_2O_3	2-7	0.5-23	227 °C<br>1:4 CO:H_2<br>conversion 2-10 %	H_2-chem/CO-chem	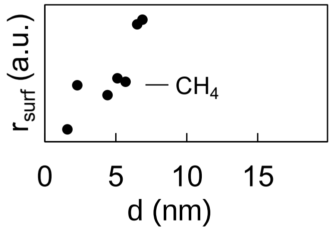

References

[1] Ertl G., Knözinger H., Schüth F. and Weitkamp J. (eds) Handbook of Heterogeneous Catalysis (Wiley-VCH Verlag, Weinheim, 2008)

[2] Bond G.C. and Hui L. The form of hydrogen chemisorption isotherms on a Pt/SiO_2 catalyst (EUROPT-1) *J. Catal.* **147**, 346-348 (1994)

[3] Shannon S.L. and Goodwin J.G. Characterization of catalytic surfaces by isotopic-transient kinetics during steady-state reaction *Chem. Rev.* **95**, 677-695 (1995)

[4] Frenkel A.I., Hills C.W., Nuzzo R.G. A view from the inside: complexity in the atomic scale ordering of supported metal nanoparticles *J. Phys. Chem B* **105**, 12689-12703 (2001)

[5] Roldan Cuenya B., Croy J.R., Mostafa S., Behafarid F., Li L., Zhang Z., Yang J.C., Wang Q., Frenkel A.I. Solving the structure of size-selected Pt nanocatalysts synthesized by inverse micelle encapsulation *J. Am. Chem. Soc.* **132**, 8747-8756 (2010)

[6] Sapelkin A.V., Bayliss S.C. Distance dependence of mean-square relative displacements in EXAFS *Phys. Rev. B* **65**, 172104 (2002)

[7] Calvin S. XAFS for everyone *CRC press* (2013)

[8] Leoni M., Confente T., Scardi P. PM2K: a flexible program implementing Whole Powder Pattern Modelling, *Z. Kristallogr. Suppl.* **23**, 249-254 (2006)

[9] Warren B.E. X-ray diffraction *Massachusetts: Addison-Wesley*, 275-298 (1969)

[10] Velterop L., Delhez R., de Keijser Th. H., Mittemeijer E.J., Reefman D. X-ray diffraction analysis of stacking and twin faults in f.c.c. metals: a revision and allowance for texture and non-uniform fault probabilities *J. Appl. Cryst.* **33**, 296-306 (2000)

[11] Estevez-Rams E., Leoni M., Scardi P., Aragon-Fernandez B., Fuess H. On the powder diffraction pattern of crystals with stacking faults *Philisophical Magazine* **83**, 4045-4057 (2003)

[12] Mears D.E. *Chem. Eng. Sci.* **26,** 1361-1366 (1971)

[13] Gierman H. *Appl. Catal*. **43**, 277-286 (1988)

[14] Wakao N., Kaguei S., Funazkri T. *Chem. Eng. Sci.* **34**, 325-336 (1979)

[15] Puncochar M., Drahos J. *Chem. Eng. Sci.* **48**, 2173-2175 (1993)

[16] Mears D.E. *J. Catal.* **20**, 127-131 (1971)

[17] Froment G.F., Bischoff K. Chemical Reactor Analysis and Design, J. Wiley & Sons, New York (1990)

[18] Specchia V. et al. *Chem. Eng. Commun.* **4**, 361-380 (1980)
